# Diagnostic performance of COVID‐19 serological assays during early infection: A systematic review and meta‐analysis of 11 516 samples

**DOI:** 10.1111/irv.12841

**Published:** 2021-02-20

**Authors:** John J. Y. Zhang, Keng Siang Lee, Chee Wui Ong, Mae Yee Chan, Li Wei Ang, Yee Sin Leo, Mark I‐Cheng Chen, David Chien Boon Lye, Barnaby Edward Young

**Affiliations:** ^1^ Yong Loo Lin School of Medicine National University of Singapore Singapore Singapore; ^2^ Bristol Medical School, Faculty of Health Sciences University of Bristol Bristol UK; ^3^ National Centre for Infectious Diseases Singapore Singapore; ^4^ Tan Tock Seng Hospital Singapore Singapore; ^5^ Lee Kong Chian School of Medicine Nanyang Technological University Singapore Singapore; ^6^ Saw Swee Hock School of Public Health National University of Singapore Singapore Singapore

**Keywords:** antibody, CLIA, COVID‐19, diagnosis, ELISA, ICA, immunoglobulin, serology

## Abstract

**Objective:**

The use of coronavirus disease 2019 (COVID‐19) serological testing to diagnose acute infection or determine population seroprevalence relies on understanding assay accuracy during early infection. We aimed to evaluate the diagnostic performance of serological testing in COVID‐19 by providing summary sensitivity and specificity estimates with time from symptom onset.

**Methods:**

A systematic search of Ovid MEDLINE, Embase, Cochrane Central Register of Controlled Trials (CENTRAL) and PubMed was performed up to May 13, 2020. All English language, original peer‐reviewed publications reporting the diagnostic performance of serological testing vis‐à‐vis virologically confirmed SARS‐CoV‐2 infection were included.

**Results:**

Our search yielded 599 unique publications. A total of 39 publications reporting 11 516 samples from 8872 human participants met eligibility criteria for inclusion in our study. Pooled percentages of IgM and IgG seroconversion by Day 7, 14, 21, 28 and after Day 28 were 37.5%, 73.3%, 81.3%, 72.3% and 73.3%, and 35.4%, 80.6%, 93.3%, 84.4% and 98.9%, respectively. By Day 21, summary estimate of IgM sensitivity was 0.872 (95% CI: 0.784‐0.928) and specificity 0.973 (95% CI: 0.938‐0.988), while IgG sensitivity was 0.913 (95% CI: 0.823‐0.959) and specificity 0.960 (95% CI: 0.919‐0.980). On meta‐regression, IgM and IgG test accuracy was significantly higher at Day 14 using enzyme‐linked immunosorbent assay (ELISA) compared to other methods.

**Conclusions:**

Serological assays offer imperfect sensitivity for the diagnosis of acute SARS‐CoV‐2 infection. Estimates of population seroprevalence during or shortly after an outbreak will need to adjust for the delay between infection, symptom onset and seroconversion.

## 1 INTRODUCTION

On March 12, 2020, the World Health Organisation (WHO) declared the outbreak of severe acute respiratory syndrome coronavirus 2 (SARS‐CoV‐2) and associated coronavirus disease 2019 (COVID‐19) as a pandemic. Unprecedented control measures have been put in place in an effort to reduce transmission, which at their peak is estimated to have covered a third of the global population.^1^, ^2^, ^3^ Despite these measures, by July 15, 2020, 13.5 million confirmed cases had been reported worldwide with 581 221 deaths—a crude case fatality rate of 4.3%.^4^ However, the true number of infections (and deaths) is likely to be substantially higher due to the large proportion of infections which are undiagnosed because of atypical, mild or absent symptoms, or unconfirmed because testing was not available.^5^, ^6^, ^7^, ^8^

Serological assays have the potential to play an important role in the surveillance of COVID‐19. The results of early seroprevalence studies have indicated that during the first wave of the COVID‐19 pandemic, up to 10% of the population of Wuhan, China may have been infected, and up to 33% in cities of other countries have experienced large outbreaks.^9^, ^10^ Serology can also be used for the diagnosis of acute infection and can form an important tool in containment strategies by identifying and linking clusters of infection.^11^ If a serological immune correlate of protection can be found, these assays may also form part of exit strategies from control measures.^12^

While serological assays have been reported to have high sensitivity and specificity for SARS‐CoV‐2 infection, this reflects diagnostic performance during convalescence.^13^ Understanding antibody kinetics during early SARS‐CoV‐2 infection is critical for assessing the accuracy of diagnostic serological results and for interpreting the results of seroprevalence studies. To address this issue, we conducted a systematic review and meta‐analysis to evaluate the diagnostic performance of serological assays in early COVID‐19, when compared to polymerase chain reaction (PCR) as the gold standard.

## 2 MATERIALS AND METHODS

This review was conducted in accordance to the Preferred Reporting Items for Systematic Reviews and Meta‐Analyses (PRISMA) guidelines.^14^

### 2.1 Search strategy and study selection

A search string comprising synonyms of “COVID‐19” and “serological assays” was applied to the following databases: Ovid MEDLINE, Embase, Cochrane Central Register of Controlled Trials (CENTRAL) and PubMed from November 1, 2019 to May 13, 2020 (Table S1). Studies were screened independently by two reviewers (JJYZ and KSL) with disagreements resolved by consensus or appeal to a third senior reviewer (BEY). Agreement between the reviewers on study inclusion was evaluated using Cohen's κ.^15^

All English language, original peer‐reviewed publications reporting the diagnostic performance of serological testing in comparison with virologically confirmed SARS‐CoV‐2 infection were included. Specific inclusion and exclusion criteria are outlined in Table S2.

### 2.2 Risk of bias assessment

The quality of included studies was assessed using QUADAS‐2 (Table S3 and Figure S1).^16^ In summary, the QUADAS‐2 tool consists of four key domains that discuss patient selection, index test, reference standard and flow of patients through the study and timing of the index tests and reference standard. Two researchers (KSL and CWO) assessed the quality of all included studies and discussed discrepancies until consensus was reached.

### 2.3 Data extraction and outcome measures

Data were extracted on the following variables: study, sample and patient details, method of diagnosis, type of blood sample and immunoassay, commercial name of test kit, cut‐off values adopted, percentage seroconversion for IgM and IgG and the time after symptom onset of serological testing. Outcome measures used were sensitivity and specificity, or true positive (TP), false negative (FN), false positive (FP) and true negative (TN) values. Prevalence, positive predictive value (PPV), negative predictive value (NPV) and F1 scores were calculated from TP, FN, FP and TN values.

### 2.4 Statistical analysis

Random effects models were used for meta‐analyses of variables and end points.^17^ Pooled proportions were computed with the inverse variance method using the variance‐stabilizing Freeman‐Tukey double arcsine transformation.^18^ Confidence intervals (CI) for individual studies were calculated using the Wilson Score confidence interval method with continuity correction. The *I* ^2^ statistic was used to present between‐study heterogeneity, where *I* ^2^ ≤ 30%, between 30% and 50%, between 50% and 75%, and ≥75% were considered to indicate low, moderate, substantial and considerable heterogeneity, respectively.^19^ *P* values for the *I* ^2^ statistic were derived from the chi‐square distribution of Cochran *Q* test. For pooling of means of numerical variables, we computed missing means and standard deviations (SDs) from medians, ranges and interquartile ranges using the methods proposed by Hozo et al and Wan et al^20^, ^21^ Standard logit confidence intervals for PPV and NPV were calculated as proposed by Mercaldo et al^22^

Bivariate summary receiver operating characteristic (SROC) curves and point estimates of sensitivity and specificity were computed with the approach proposed by Reitsma et al,^23^ using a linear mixed model with known variances of the random effects. Bivariate meta‐regression with additional likelihood‐ratio tests was performed to evaluate for any significant effects of covariates. In addition, univariate meta‐analysis was also done using random effect estimation with the DerSimonian‐Laird method to produce pooled diagnostic odds ratios.^24^

Publication bias was assessed using funnel plots and Egger's regression test, based on a weighted linear regression of the treatment effect on its standard error.^25^, ^26^

All statistical analyses were performed using R software version 3.4.3 , with the packages *meta* and *mada*.^27^, ^28^ *P* values less than .05 were considered statistically significant.

## 3 RESULTS

### 3.1 Study characteristics

Our search yielded 599 unique publications, of which 72 were reviewed in full text. A total of 39 publications reporting 11 516 samples taken from 8872 human participants met criteria for inclusion in our study (Figure S2).^10^, ^29^, ^30^, ^31^, ^32^, ^33^, ^34^, ^35^, ^36^, ^37^, ^38^, ^39^, ^40^, ^41^, ^42^, ^43^, ^44^, ^45^, ^46^, ^47^, ^48^, ^49^, ^50^, ^51^, ^52^, ^53^, ^54^, ^55^, ^56^, ^57^, ^58^, ^59^, ^60^, ^61^, ^62^, ^63^, ^64^, ^65^, ^66^ Reliability of study selection between observers was substantial at both the title and abstract screening stage (Cohen's κ = 0.91) and the full‐text review stage (Cohen's κ = 0.94).

Of the 39 publications, 55 distinct studies were conducted. Studies were regarded as distinct if they had different study designs, evaluated different cohorts or analysed different test kits with results reported separately. Of the 55 studies, 37 were retrospective and 18 were prospective. 24 were case‐control studies, 19 were case series of COVID‐19 patients, and 12 were cohort studies including both COVID‐19 and non‐COVID‐19 participants. The majority of studies were from China (n = 34, 61.8%), followed by Italy (n = 7, 12.7%) and France (n = 6, 10.9%). There were three studies from Hong Kong and one study each from the United States, Germany, Sweden, Japan and Taiwan. Characteristics of included studies are summarized in Table S4.

The type of immunoassay used was specified in 47 studies. Among these, 18 used immunochromatographic assay (ICA), 15 used enzyme‐linked immunosorbent assay (ELISA), 13 used chemiluminescent immunoassay (CLIA) and one used dry fluorescence. Forty eight studies tested both IgM and IgG, four studies tested IgM only, two studies tested IgG only and one study tested IgA only.

### 3.2 Quality assessment with QUADAS‐2

Among the 39 publications, the proportion of studies with low, high and unclear risk of bias and concerns regarding applicability are summarized in Table S3 and Figure S1. More than half of the studies were found to be at high risk of bias under the domains “Patient Selection and Index Test.” The main causes for high risk of bias were due to non‐cohort study designs and cut‐off values not being reported for the respective serological assays.

### 3.3 Characteristics of patients and controls

Of the 11 516 samples analysed in total, 5743 were taken from laboratory‐diagnosed COVID‐19 patients, 5265 were from healthy controls and 508 were from patients infected with human coronaviruses (229E, HKU1, NL63, OC43 and SARS‐CoV (using convalescent samples)), influenza A and B, and other respiratory pathogens.

A total of 5743 serum samples were acquired from 3630 patients with COVID‐19. 27 studies reported patient gender, of which 50.5% were females (962 out of 1906) and 49.5% were males (944 out of 1906). Pooled mean age of patients across the 22 studies that reported mean and SD of age was 53.8 years (95% CI: 49.7‐57.8). The most commonly reported comorbidities were diabetes mellitus, malignancy and hypertension (pooled percentages of 11.6%, 13.4% and 20.0% reported across seven, five and four studies, respectively).

### 3.4 IgM Seroconversion

**FIGURE 1:**
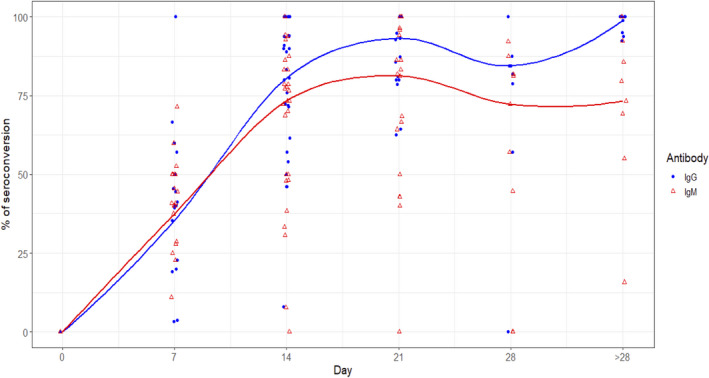
Graph of IgM and IgG seroconversion against time from symptom onset

A total of 44 studies reported percentage of IgM seroconversion across 4026 samples, with 30 studies specifying the time of serological testing. 20 studies performed serological testing within 28 days of symptom onset while 10 performed testing within a time interval that extended beyond 28 days up to Day 50. Pooled percentage of IgM seroconversion was 75.3% (95% CI: 69.7‐80.6; Figure S3). Study heterogeneity was considerable (*I* ^2^ = 91.5%, *P* < .0001). Pooled percentages of IgM seroconversion by Day 7, 14, 21, 28 and after Day 28 were 37.5%, 73.3%, 81.3%, 72.3% and 73.3%, respectively (Table 1 and Figure 1). No evidence of publication bias for IgM seroconversion was identified on Egger's regression test (*P* = .69; Figure S4). Subgroup meta‐analysis identified no significant difference in IgM seroconversion rates between the various types of immunoassay (*P* = .44; Figure S5).

**TABLE 1:** Percentages of IgM and IgG seroconversion by weeks from symptom onset

Outcome	No. of studies reporting variable	No. of samples analysed	Pooled percentage of samples (95% CI)	*I* ^2^ (%)	*P* value from χ^2^ test
IgM seroconversion	44	4026	75.3 (69.7‐80.6)	91.5	<.0001
By Day 7	19	491	37.5 (30.9‐44.4)	43.0	.0246
By Day 14	26	706	73.3 (64.7‐81.2)	76.4	<.0001
By Day 21	17	349	81.3 (69.7‐91.1)	79.1	<.0001
By Day 28	8	213	72.3 (48.8‐91.7)	84.3	<.0001
After Day 28	7	179	73.3 (51.5‐90.8)	87.1	<.0001
IgG seroconversion	43	4211	85.8 (78.6‐92.0)	96.4	<.0001
By Day 7	19	486	35.4 (23.9‐47.7)	82.5	<.0001
By Day 14	24	686	80.6 (70.0‐89.7)	86.7	<.0001
By Day 21	16	337	93.3 (86.1‐98.4)	66.7	<.0001
By Day 28	7	212	84.4 (68.1‐96.4)	79.3	<.0001
After Day 28	7	181	98.9 (95.6‐100.0)	12.8	.3318

### 3.5 IgG Seroconversion

A total of 43 studies reported percentage of IgG seroconversion across 4211 samples, with 27 studies specifying the time of serological testing. 17 studies performed serological testing within 28 days of symptom onset while 10 performed testing within a time interval that extended beyond 28 days up to Day 50. Pooled percentage of IgG seroconversion was 85.8% (95% CI: 78.6‐92.0). Study heterogeneity was considerable (*I* ^2^ = 96.4%, *P* < .0001; Figure S6). Pooled percentages of IgG seroconversion by Day 7, 14, 21, 28 and after Day 28 were 35.4%, 80.6%, 93.3%, 84.4% and 98.9%, respectively (Table 1 and Figure 1). No evidence of publication bias for IgG seroconversion was identified on Egger's regression test (*P* = .70; Figure S4). Subgroup meta‐analysis identified no significant difference in IgG seroconversion rates between the various types of immunoassay (*P* = .56; Figure S7).

### 3.6 Diagnostic accuracy of IgM testing

**FIGURE 2:**
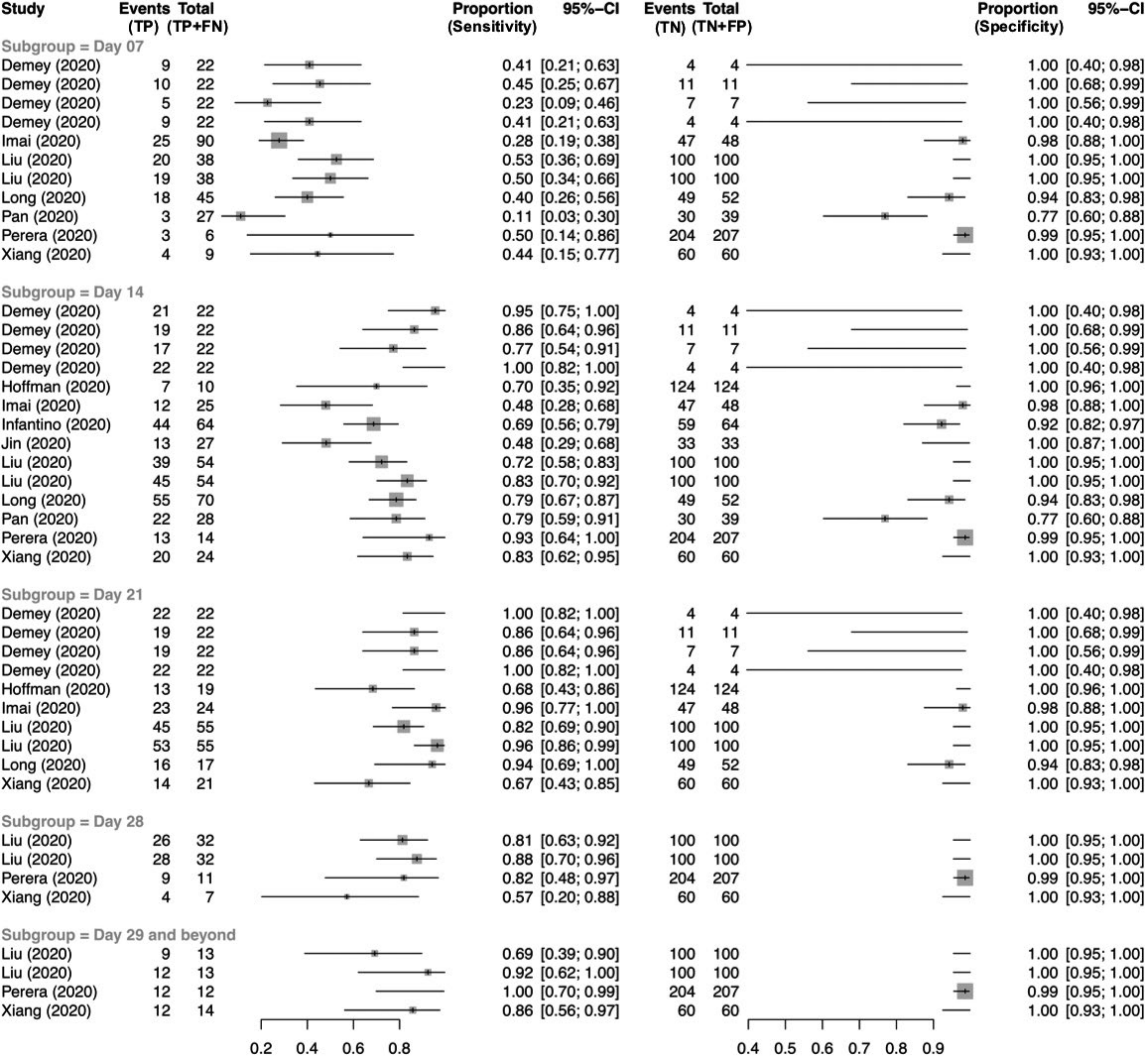
Coupled forest plot showing sensitivity and specificity of IgM testing stratified by time from symptom onset

TP, FN, TN and FP values for IgM testing were reported in 24 studies. Figure 2 demonstrates a coupled forest plot of sensitivity and specificity values reported across all 24 studies, stratified by time from symptom onset. On univariate meta‐analysis, diagnostic odds ratio for IgM testing was 41.4 (95% CI: 16.1‐106.6). Study heterogeneity was negligible (*I* ^2^ = 0.0%, *P* = .54).

Using a bivariate SROC curve, the overall summary estimates of IgM sensitivity and specificity were 0.727 (95% CI: 0.658‐0.786) and 0.918 (95% CI: 0.805‐0.969), respectively (Figure S8). Sensitivity and specificity values of IgM testing by Day 7, 14 and 21 are shown in Table 2. By Day 21, summary estimate of IgM sensitivity was 0.872 (95% CI: 0.784‐0.928) and specificity was 0.973 (95% CI: 0.938‐0.988). Prevalence, PPV, NPV and F1 scores of studies reporting IgM testing stratified by time from symptom onset are shown in Table S5.

**TABLE 2:** Summary of pooled diagnostic performance using IgM, IgG and combined IgM and IgG assays

Serological test and time of test	No. of studies reporting variable	No. of samples analysed	Diagnostic odds ratio	*I* ^2^ (%)	*P* value from χ^2^ test	Sensitivity (95% confidence interval)	Specificity (95% confidence interval)	AUC
IgM assay	24	3719	41.4 (16.1‐106.6)	0.0	.541	0.727 (0.658‐0.786)	0.918 (0.805‐0.969)	0.826
By Day 7	11	973	15.3 (4.4‐53.3)	0.0	.617	0.381 (0.300‐0.469)	0.962 (0.917‐0.983)	0.696
By Day 14	14	1311	89.5 (38.7‐206.9)	0.0	.558	0.752 (0.674‐0.816)	0.962 (0.921‐0.982)	0.913
By Day 21	10	789	349.5 (142.2‐859.1)	0.0	.825	0.872 (0.784‐0.928)	0.973 (0.938‐0.988)	0.976
IgG assay	27	6452	87.4 (31.0‐246.2)	13.6	.264	0.788 (0.684‐0.865)	0.948 (0.882‐0.978)	0.922
By Day 7	11	973	10.1 (2.5‐40.3)	0.0	.444	0.317 (0.200‐0.463)	0.951 (0.875‐0.981)	0.730
By Day 14	14	1311	119.9 (33.8‐424.6)	0.0	.894	0.793 (0.660‐0.883)	0.959 (0.902‐0.983)	0.948
By Day 21	10	789	304.2 (129.7‐713.5)	0.0	.485	0.913 (0.823‐0.959)	0.960 (0.919‐0.980)	0.979
IgM or IgG assay	16	3079	51.8 (20.9‐128.4)	12.5	.310	0.740 (0.636‐0.823)	0.936 (0.887‐0.965)	0.924
By Day 7	5	641	15.6 (1.2‐207.2)	2.3	.393	0.409 (0.224‐0.624)	0.953 (0.763‐0.992)	0.719
By Day 14	6	698	100.8 (26.4‐384.4)	24.3	.252	0.849 (0.700‐0.931)	0.946 (0.801‐0.987)	0.944
By Day 21	4	451	1079.1 (269.4‐4323.1)	0.0	.541	0.949 (0.900‐0.974)	0.975 (0.903‐0.994)	0.952

On meta‐regression, specificity of IgM testing by Day 14 was significantly higher using ELISA than CLIA (*P* = .006), with similar sensitivity values (*P* = .11). The likelihood‐ratio test further suggested a significant difference in test accuracy values with ELISA versus CLIA (χ^2^ = 10.55, *P* = .0051). ELISA also produced a significantly higher specificity than ICA in IgM testing by Day 14 (*P* = .014), with similar sensitivity values (*P* = .83). On further comparison using the likelihood‐ratio test, however, the difference in diagnostic accuracy between the two tests was only close to statistical significance (*P* = .071).

### 3.7 Diagnostic accuracy of IgG testing

**FIGURE 3:**
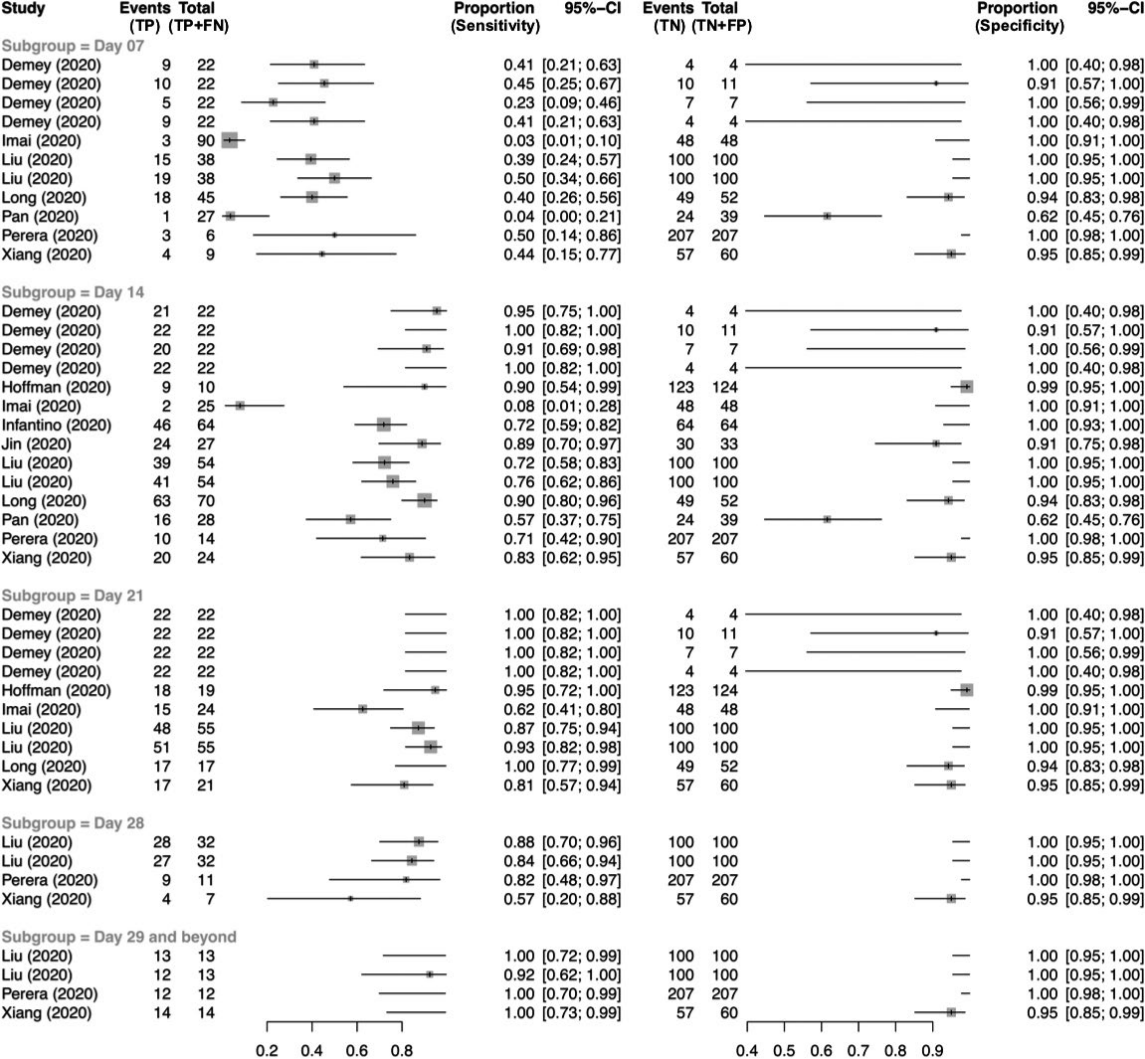
Coupled forest plot showing sensitivity and specificity of IgG testing stratified by time from symptom onset

TP, FN, TN and FP values for IgG testing were reported in 27 studies. Figure 3 demonstrates a coupled forest plot of sensitivity and specificity values reported across all 27 studies. On univariate meta‐analysis, diagnostic odds ratio for IgG testing was 87.4 (95% CI: 31.0‐246.2). Study heterogeneity was insignificant (*I* ^2^ = 13.6%, *P* = .26).

Using a bivariate SROC curve, the summary estimates of IgG sensitivity and specificity were 0.788 (95% CI: 0.684‐0.865) and 0.948 (95% CI: 0.882‐0.978), respectively (Figure S9). Sensitivity and specificity values of IgG testing by Day 7, 14 and 21 are shown in Table 2. By Day 21, summary estimate of IgG sensitivity was 0.913 (95% CI: 0.823‐0.959) and specificity was 0.960 (95% CI: 0.919‐0.980). Prevalence, PPV, NPV and F1 scores of studies reporting IgG testing stratified by time from symptom onset are shown in Table S6.

Meta‐regression demonstrated that IgG testing performed by Day 14 using ELISA had a higher specificity than ICA that was close to statistical significance (*P* = .056), with comparable sensitivity values (*P* = .19).

### 3.8 Combined value of IgM and IgG testing

TP, FN, FP and TN values for serological testing using IgM or IgG were reported in 16 studies. On univariate meta‐analysis, diagnostic odds ratio for testing using IgM or IgG was 51.8 (95% CI: 20.9‐128.4). Study heterogeneity was negligible (*I* ^2^ = 12.5%, *P* = .31).

Using a bivariate SROC curve, the summary estimates of sensitivity and specificity were 0.740 (95% CI: 0.636‐0.823) and 0.936 (95% CI: 0.887‐0.965), respectively (Figure S10). Sensitivity and specificity values of testing for IgM or IgG by Day 7, 14 and 21 are shown in Table 2. By Day 21, summary estimate of sensitivity using IgM or IgG testing was 0.949 (95% CI: 0.900‐0.974) and specificity was 0.975 (95% CI: 0.903‐0.994).

On meta‐regression, ELISA was found to have higher specificity values than CLIA (*P* = .011), with similar sensitivity values (*P* = .67). The likelihood‐ratio test further suggested a significant difference in test accuracy values with CLIA versus ELISA (χ^2^ = 6.62, *P* = .036). ELISA also demonstrated higher specificity values than ICA (*P* = .021), with comparable sensitivity values (*P* = .51). On further comparison using the likelihood‐ratio test, however, the difference in diagnostic accuracy between the two tests was only close to statistical significance (*P* = .072).

## 4 DISCUSSION

Our meta‐analysis indicated that the overall sensitivity and specificity of serological testing for the diagnosis of COVID‐19 were 0.727 (95% CI: 0.658‐0.786) and 0.918 (95% CI: 0.805‐0.969) for IgM, and 0.788 (95% CI: 0.684‐0.865) and 0.948 (95% CI: 0.882‐0.978) for IgG, respectively. The performance of serological testing was strongly influenced by the duration of infection: sensitivity of IgM increased from 0.381 (95% CI: 0.300‐0.469) at Day 7 to 0.872 (95% CI: 0.784‐0.928) at Day 21, while sensitivity of IgG increased from 0.317 (95% CI: 0.200‐0.463) at Day 7 to 0.913 (95% CI: 0.823‐0.959) at Day 21. Combined testing for IgM and IgG offered similar results to IgG alone with a sensitivity of 0.949 (95% CI: 0.900‐0.974) at Day 21.

Similarly, rates of IgM and IgG seroconversion were low in the first week of infection at only 37.5% and 35.4%, respectively. For IgM, this rose to 81.3% at Day 21 then declined to 73.3% after Day 28, while for IgG, this rose to 93.3% at Day 21 and 98.9% beyond Day 28. This delayed time to seroconversion indicates that the serological assays included in this review can only play a limited role in the early diagnosis of COVID‐19. An early, accurate diagnosis is important for clinical management and necessary for prompt isolation of the infected individual, and contact tracing and quarantine of exposed contacts. An ELISA‐based IgG serological test beyond Day 28 from symptom onset will yield accurate results, but such a delayed diagnosis is outside the window for targeting effective control measures. The low sensitivity of IgM reported in our meta‐analysis may reflect serological tests which have been calibrated to offer high specificity (>95% from Day 7 to 21) at the cost of sensitivity in order to overcome the lower affinity and relative non‐specific nature of IgM binding.

Improving IgM sensitivity with attendant lower specificity may offer acceptable accuracy in an outbreak scenario where the prevalence of COVID‐19 is high. However, this situation is likely to change. An unintended consequence of physical efforts to reduce SARS‐CoV‐2 transmission is historically low rates of influenza infections in Singapore, Australia and New Zealand.^67^, ^68^ However, with the easing of lockdowns and as winter approaches in the Northern Hemisphere, circulation of influenza and other respiratory viruses is likely to increase. In this situation, the prevalence of COVID‐19 among symptomatic individuals will decline assuming there is a significant additional number of acute respiratory infections from other causes. Tests with inadequate specificity will hence yield more false positive results and lower positive predictive values. Furthermore, while cross‐reactivity with other human coronaviruses was not evident from the diagnostic performance of assays included in our review, this may become an issue with less specific but more sensitive testing.

An additional question is how these tests will perform in the event of further waves of COVID‐19 where a substantial proportion of the population may already have been infected. IgM can persist for months, reducing the specificity of serological diagnosis.^69^ With wide variation in seroprevalence between different regions of a country, age groups and contact clusters, the epidemiological context for test interpretation becomes extremely complex. Ongoing seroprevalence studies will also require repeated iterations given the delay in time to seroconversion.

Our findings are in accordance with those from two previous systematic reviews. Lisboa Bastos et al reviewed 40 studies that investigated the diagnostic performance of serological testing for COVID‐19 and concluded that current evidence does not support the continued use of existing point‐of‐care serological tests.^70^ Similarly, a Cochrane Database review by Deeks et al found that the sensitivity of antibody tests is too low in the first week since symptom onset to have a primary role for the diagnosis of COVID‐19, and at present, there exists very little evidence beyond 35 days after symptom onset.^71^ In addition to confirming the above findings with a more updated search, we present a meta‐regression analysis that suggested superiority of ELISA over ICA and CLIA in diagnosing COVID‐19 infection.

There were several limitations of our study. First, only studies published in English were included, which may have introduced selection bias. Second, a majority of studies were from Chinese institutions (61.8%); therefore, this may not accurately reflect the performance of serological testing in non‐Asian continents. Third, the diagnostic performance of the various serological tests may have been influenced by clinical and host factors, including the severity of disease, age, comorbidities and medications. Clinical symptoms and treatment details were scarcely reported in the included studies; therefore, our analysis was unable to adjust for these factors. Fourth, quantification of the amount of IgM or IgG, and not just a categorical positive or negative result is important to analyse. However, there was too much variability in study designs and testing methods to assess for this. Fifth, there was a paucity of data for serological tests done beyond 28 days of disease onset; hence, further studies would be needed to elucidate the diagnostic performance of antibody testing in individuals beyond 28 days. Finally, despite RT‐PCR being deemed to be the gold standard for COVID‐19 diagnosis, it may itself have imperfect sensitivity, especially when the viral load is low or when patients are tested late in the course of the infection.^72^, ^73^, ^74^ This leads to possible false‐negative PCR results and should be considered as a limitation of our findings.

## 5 CONCLUSIONS

Our study demonstrated that the utility of serological assays is limited to IgG more than 14‐21 days after symptom onset, when most infected individuals have seroconverted and high sensitivity and specificity values are attained. Diagnostic testing is hence likely to continue to require virological confirmation such as PCR, while seroprevalence studies will significantly underestimate the proportion of a population infected if conducted too early in the epidemic curve. Longer‐term studies would be beneficial to improve our understanding of the diagnostic performance of serological testing for COVID‐19 in other epidemiological contexts such as endemic infection or future epidemics.

## CONFLICT OF INTEREST

The authors declare no competing interests.

## AUTHOR CONTRIBUTIONS

**John J. Y. Zhang:** Data curation (equal); Formal analysis (equal); Investigation (equal); Methodology (equal); Project administration (equal); Resources (equal); Software (equal); Validation (equal); Visualization (equal); Writing‐original draft (equal); Writing‐review & editing (equal). **Keng Siang Lee:** Data curation (equal); Investigation (equal); Methodology (equal); Project administration (equal); Resources (equal); Validation (equal); Visualization (equal); Writing‐original draft (equal); Writing‐review & editing (equal). **Chee Wui Ong:** Data curation (equal); Investigation (equal); Methodology (equal); Project administration (equal); Resources (equal); Validation (equal); Visualization (equal); Writing‐review & editing (equal). **Mae Yee Chan:** Data curation (equal); Investigation (equal); Methodology (equal); Project administration (equal); Resources (equal); Validation (equal); Visualization (equal); Writing‐review & editing (equal). **Li Wei Ang:** Formal analysis (equal); Investigation (equal); Methodology (equal); Software (equal); Validation (equal); Writing‐review & editing (equal). **Yee Sin Leo:** Funding acquisition (equal); Investigation (equal); Methodology (equal); Resources (equal); Supervision (equal); Validation (equal); Visualization (equal); Writing‐review & editing (equal). **Mark I‐Cheng Chen:** Funding acquisition (equal); Investigation (equal); Methodology (equal); Resources (equal); Supervision (equal); Validation (equal); Writing‐review & editing (equal). **David Chien Boon Lye:** Funding acquisition (equal); Investigation (equal); Methodology (equal); Resources (equal); Supervision (equal); Validation (equal); Writing‐review & editing (equal). **Barnaby Edward Young:** Conceptualization (equal); Data curation (equal); Formal analysis (equal); Funding acquisition (equal); Investigation (equal); Methodology (equal); Resources (equal); Supervision (equal); Validation (equal); Visualization (equal); Writing‐review & editing (equal).

### PEER REVIEW

The peer review history for this article is available at https://publons.com/publon/10.1111/irv.12841.

## Supporting information

Supporting Information

## Data Availability

The data that support the findings of this study are available in the supplementary material of this article.
