# Supplementary material for: Diagnostic performance of COVID‐19 serological assays during early infection: A systematic review and meta‐analysis of 11 516 samples
Source: Influenza Other Respir Viruses. 2021 Feb 20;15(4):529–38. doi: 10.1111/irv.12841 (PMC8013346; doi:10.1111/irv.12841)
Supplement: Supplementary file 1 — Supporting Information [file IRV-15-529-s001.pdf]

Online-only Supplementary Document

Supplementary Tables

Table S1. Full search phrases used for the 4 respective databases.

Table S2. Inclusion and exclusion criteria used to assess eligibility of studies.

Table S3. QUADAS-2 assessment results.

Table S4. Summary of all 55 included studies across 39 publications.

Table S5. Summary of diagnostic performance for all included studies using IgM testing.

Table S6. Summary of diagnostic performance for all included studies using IgG testing.

Supplementary Figures

Figure S1. Bar charts demonstrating quality of included studies assessed using QUADAS-2.

Figure S2. PRISMA flow diagram showing study selection and inclusion.

Figure S3. Pooled percentage of IgM seroconversion across all included studies.

Figure S4. Funnel plots studies reporting IgM seroconversion (top) and IgG seroconversion (bottom).

Figure S5. Subgroup analysis of IgM seroconversion percentages across studies stratified by type of immunoassay.

Figure S6. Pooled percentage of IgG seroconversion across all included studies.

Figure S7. Subgroup analysis of IgG seroconversion percentages across studies stratified by type of immunoassay.

Figure S8. Bivariate summary receiver operating characteristic curve for IgM assay.

Figure S9. Bivariate summary receiver operating characteristic curve for IgG assay.

Figure S10. Bivariate summary receiver operating characteristic curve for testing using IgM or IgG.

Table S1. Full search phrases used for the 4 respective databases.

**Pubmed**		502 articles
(("Coronavirus"[MeSH Terms]) OR ("Coronavirus Infections"[MeSH Terms]) OR (COVID-19[Text Word]) OR (coronavirus disease 2019[Text Word]) OR (2019-nCoV[Text Word]) OR (novel coronavirus[Text Word]) OR (SARS-CoV-2[Text Word]) OR (severe acute respiratory syndrome coronavirus 2[Text Word]) OR (NCIP[Text Word]) OR (novel coronavirus infected pneumonia[Text Word])) AND ((“Serology”[MeSH Terms]) OR (serolog*[Text Word]) OR (“Antibodies”[MeSH Terms]) OR (antibod*[Text Word]) OR (“Immunoglobulins”[MeSH Terms]) OR (immunoglobulin*[Text Word]) OR (“Immunoglobulin G”[MeSH Terms]) OR (IgG[Text Word]) OR (“Immunoglobulin M”[MeSH Terms]) OR (IgM[Text Word]) OR (“Immunoglobulin A”[MeSH Terms]) OR (IgA[Text Word]) OR (polyclonal[Text Word]) OR (“Antibodies, Monoclonal”[MeSH Terms]) OR (monoclonal[Text Word]) OR (“Enzyme-linked Immunosorbent Assay”[MeSH Terms]) OR (enzyme-linked immunosorbent assay[Text Word]) OR (enzyme linked immunosorbent assay[Text Word]) OR (ELISA[Text Word]) OR (EIA[Text Word]) OR (lateral-flow immunoassay[Text Word]) OR (lateral flow immunoassay[Text Word]) OR (colloidal-gold lateral-flow immunoassay[Text Word]) OR (LFIA[Text Word]) OR (fluorescence immunochromatographic assay[Text Word]) OR (chemiluminescence immunoassay[Text Word]) OR (chemi-luminescence immunoassay[Text Word]) OR (CLIA[Text Word]) OR (chemiluminescent*[Text Word]) OR (“Immunoassay”[MeSH Terms]) OR (immunoassay[Text Word]) OR (immunosorbent*[Text Word]) OR (“Fluorescent Antibody Technique”[MeSH Terms]) OR (immunofluorescen*[Text Word]) OR (immunoblot[Text Word]) OR (“Blotting, Western”[MeSH Terms]) OR (western blot*[Text Word]))		
**Ovid MEDLINE**		190 articles
COVID-19 concept		
1	Exp Coronavirus/	
2	Exp Coronavirus Infections/	
3	COVID-19.tw.	
4	Coronavirus disease 2019.tw.	
5	2019-nCoV.tw.	
6	Novel coronavirus.tw.	

7	SARS-CoV-2.tw.
8	Severe acute respiratory syndrome coronavirus 2.tw.
9	NCIP.tw.
10	Novel coronavirus infected pneumonia.tw.
11	1 or 2 or 3 or 4 or 5 or 6 or 7 or 8 or 9 or 10
Immunoglobulin diagnosis concept	
12	Exp serology/
13	serolog*.tw
14	exp Antibodies/
15	Antibod*.tw
16	Exp Immunoglobulins/
17	immunoglobulin*.tw
18	Exp Immunoglobulin G/
19	IgG.tw
20	Exp Immunoglobulin M/
21	IgM.tw
22	Exp Immunoglobulin A/
23	IgA.tw
24	Polyclonal.tw

25	Exp Antibodies, Monoclonal/
26	Monoclonal.tw
27	Exp Enzyme-linked Immunosorbent Assay/
28	Enzyme-linked immunosorbent assay.tw
29	Enzyme linked immunosorbent assay.tw
30	ELISA.tw
31	EIA.tw
32	Lateral flow immunoassay.tw
33	Lateral-flow immunoassay.tw
34	Colloidal-gold lateral-flow immunoassay.tw
35	LFIA.tw
36	Fluorescence immunochromatographic assay.tw
37	Chemiluminescence immunoassay.tw
38	Chemi-luminescence immunoassay.tw
39	CLIA.tw
40	Chemiluminescen*.tw
41	Exp Immunoassay/
42	Immunoassay.tw
43	Immunosorben*.tw

44	Exp Fluorescent Antibody Technique/	
45	Immunofluorescen*.tw	
46	Immunoblot*.tw	
47	Exp Blotting, Western/	
48	Western blot*.tw	
49	12 or 13 or 14 or 15 or 16 or 17 or 18 or 19 or 20 or 21 or 22 or 23 or 24 or 25 or 26 or 27 or 28 or 29 or 30 or 31 or 32 or 33 or 34 or 35 or 36 or 37 or 38 or 39 or 40 or 41 or 42 or 43 or 44 or 45 or 46 or 47 or 48	
Combined concepts		
50	11 and 49	
51	Limit 50 to dt=20191101-20200513 [November 1st, 2019 to May 13th, 2020]	
**Embase**		182 articles
COVID-19 concept		
1	Exp Coronavirinae/	
2	COVID-19.tw.	
3	Coronavirus disease 2019.tw.	
4	2019-nCoV.tw	
5	Novel coronavirus.tw	
6	SARS-CoV-2.tw	
7	Severe acute respiratory syndrome coronavirus 2.tw.	

8	NCIP.tw.
9	Novel coronavirus infected pneumonia.tw
10	1 or 2 or 3 or 4 or 5 or 6 or 7 or 8 or 9
Convalescent antibody concept	
11	Exp serology/
12	serolog*.tw
13	exp Antibody/
14	Antibod*.tw
15	Exp Immunoglobulin G/ or exp immunoglobulin A/ or exp immunoglobulin M/ or exp immunoglobulin/
16	immunoglobulin*.tw
17	IgG.tw
18	IgM.tw
19	IgA.tw
20	Exp polyclonal antibody/
21	Polyclonal.tw
22	Exp Monoclonal antibody/
23	Monoclonal.tw
24	Exp Enzyme linked immunosorbent assay/
25	Enzyme-linked immunosorbent assay.tw

26	Enzyme linked immunosorbent assay.tw
27	ELISA.tw
28	EIA.tw
29	Lateral flow immunoassay.tw
30	Lateral-flow immunoassay.tw
31	Colloidal-gold lateral-flow immunoassay.tw
32	LFIA.tw
33	Fluorescence immunochromatographic assay.tw
34	Exp Chemiluminescence immunoassay/
35	Chemiluminescence immunoassay.tw
36	Chemi-luminescence immunoassay.tw
37	CLIA.tw
38	Exp Chemiluminescence/
39	Chemiluminescen*.tw
40	Exp Immunoassay/
41	Immunoassay.tw
42	Exp Immunosorbent/
43	Immunosorbent.tw
44	Exp Fluorescent Antibody Technique/

45	Immunofluorescen*.tw
46	Exp Immunoblotting/
47	immunoblot*.tw
48	Exp Western blotting/
49	Western blot*.tw
50	11 or 12 or 13 or 14 or 15 or 16 or 17 or 18 or 19 or 20 or 21 or 22 or 23 or 24 or 25 or 26 or 27 or 28 or 29 or 30 or 31 or 32 or 33 or 34 or 35 or 36 or 37 or 38 or 39 or 40 or 41 or 42 or 43 or 44 or 45 or 46 or 47 or 48 or 49
Combined concepts	
51	10 and 50
52	Limit 51 to dd=20191101-20200513 [November 1st, 2019 to May 13th, 2020]
**Cochrane Controlled Register of Trials CENTRAL**	33 articles
COVID-19 concept	
1	MeSH descriptor: [Coronavirus] explode all trees
2	(COVID-19):ti,ab,kw
3	(Coronavirus disease 2019):ti,ab,kw
4	(2019 nCoV):ti,ab,kw
5	(Novel coronavirus):ti,ab,kw
6	(SARS-CoV-2):ti,ab,kw
7	(Severe acute respiratory syndrome coronavirus 2):ti,ab,kw

8	(NCIP):ti,ab,kw
9	(Novel coronavirus infected pneumonia):ti,ab,kw
10	#1 OR #2 OR #3 OR #4 OR #5 OR #6 OR #7 OR #8 OR #9
Convalescent antibody concept	
11	MeSH descriptor: [Serology] explode all trees
12	(Serolog*):ti,ab,kw
13	MeSH descriptor: [Antibodies] explode all trees
14	(Antibod*):ti,ab,kw
15	MeSH descriptor: [Immunoglobulins] explode all trees
16	(Immunoglobulin*):ti,ab,kw
17	(IgG):ti,ab,kw
18	(IgM):ti,ab,kw
19	(IgA):ti,ab,kw
20	(Polyclonal*):ti,ab,kw
21	MeSH descriptor: [Antibodies, Monoclonal] explode all trees
22	(Monoclonal*):ti,ab,kw
23	MeSH descriptor: [Enzyme-Linked Immunosorbent Assay] explode all trees
24	(enzyme-linked immunosorbent assay):ti,ab,kw
25	(enzyme linked immunosorbent assay):ti,ab,kw

26	(ELISA):ti,ab,kw
27	(EIA):ti,ab,kw
28	(Lateral flow immunoassay):ti,ab,kw
29	(Lateral-flow immunoassay):ti,ab,kw
30	(Colloidal-gold lateral-flow immunoassay):ti,ab,kw
31	(LFIA):ti,ab,kw
32	(Fluorescence immunochromatographic assay):ti,ab,kw
33	(Chemiluminescence immunoassay):ti,ab,kw
34	(Chemi-luminescence immunoassay):ti,ab,kw
35	(CLIA):ti,ab,kw
36	MeSH descriptor: [Luminescence] explode all trees
37	(Chemiluminescen*):ti,ab,kw
38	MeSH descriptor: [Immunoassay] explode all trees
39	(Immunoassay):ti,ab,kw
40	MeSH descriptor: [Immunosorbents] explode all trees
41	(Immunosorben*l):ti,ab,kw
42	MeSH descriptor: [Fluorescent Antibody Technique] explode all trees
43	(Immunofluorescen*):ti,ab,kw
44	MeSH descriptor: [Immunoblotting] explode all trees

45	(Immunoblot*):ti,ab,kw
46	MeSH descriptor: [Blotting, Western] explode all trees
47	(western blot*):ti,ab,kw
48	#11 OR #12 OR #13 OR #14 OR #15 OR #16 OR #17 OR #18 OR #19 OR #20 OR #21 OR #22 OR #23 OR #24 OR #25 OR #26 OR #27 OR #28 OR #29 OR #30 OR #31 OR #32 OR #33 OR #34 OR #35 OR #36 #37 OR #38 OR #39 OR #40 OR #41 OR #42 OR #43 OR #44 OR #45 OR #46 OR #47
Combined concepts	
49	#10 AND #48
50	#49 with Cochrane Library publication date in The last 9 months

Table S2. Inclusion and exclusion criteria used to assess eligibility of studies.

Inclusion criteria	Exclusion criteria
• Any peer-reviewed publication reporting the diagnostic performance of rapid serological assays in patients with COVID-19<br>• Diagnosis of COVID-19 made with virologic testing<br>• At least 5 human samples from patients of any age	• Not written in English<br>• No original research data e.g. narrative and systematic reviews, editorials, commentaries, opinion papers, letters[a], education papers, conference abstracts, protocols, reports, theses or book chapters<br>• Case reports and case series with fewer than 5 patients reported<br>• Non-human samples (e.g. murine, porcine studies)<br>• Purely seroepidemiological with no outcomes of diagnostic test accuracy

[a]Letters to Editors with original research data reported were included.

Table S3. QUADAS-2 assessment results.

	Risk of Bias					Applicability Concerns		
Study	Patient Selection	Index Test	Reference Standard	Flow and Timing		Patient Selection	Index Test	Reference Standard
Bryan (2020)	☹	☺	☺	☺		☺	☺	☺
Cai (2020)	☹	☺	☺	☺		☺	☺	☺
Cassaniti (2020)	☹	☹	☺	☺		☺	☺	☺
Chen (2020)	☹	☺	☺	?		☺	☺	☺
Demey (2020)	☹	☹	☺	☺		☺	☺	☺
Dohla (2020)	☺	☹	☺	☺		☺	☺	☺
Du (2020)	☺	☺	☺	☺		☺	☺	☺
Fu (2020)	☹	☺	☺	☺		☺	☺	☺
Gao (2020)	☹	☹	☺	☺		☺	☺	☺
Guo (2020)	☺	☺	☺	☺		☺	☺	☺
Hoffman (2020)	☹	☹	☺	☺		☺	☺	☺
Hou (2020)	☺	☺	☺	☺		☺	☺	☺
Imai (2020)	☹	☹	☺	☺		☺	☺	☺
Infantino (2020)	☹	☺	☺	☺		☺	☺	☺
Jin (2020)	☹	☺	☺	☺		☺	☺	☺
Lee (2020)	☹	☹	☺	☺		☺	☺	☺
Li (2020)	☹	☹	☺	☺		☺	☺	☺
Liu (2020)	☹	☹	☺	☺		☺	☺	☺
Long (2020)	☹	☹	☺	☺		☺	☺	☺
Padoan (2020a)	☹	☺	☺	☺		☺	☺	☺
Padoan (2020b)	☺	☺	☺	☺		☺	☺	☺
Pan (2020)	☹	☹	☺	☺		☺	☺	☺
Perera (2020)	☹	☺	☺	☺		☺	☺	☺
Qu (2020)	☹	☺	☺	☺		☺	☺	☺
Shen (2020)	☺	☹	☺	☺		☺	☺	☺

Shi (2020)	☹	☹	☺	☺		☺	☺	☺
Solodky (2020)	☺	☹	☺	☺		☹	☺	☺
Spicuzza (2020)	☹	☹	☺	☺		☺	☺	☺
To (2020)	☹	☹	☺	☺		☺	☺	☺
Wang (2020)	☹	☹	☺	☺		☺	☺	☺
Wu (2020)	☺	☹	☺	☺		☺	☺	☺
Xiang (2020)	☹	☹	☺	☺		☺	☺	☺
Xie (2020)	☺	☺	☺	☺		☺	☺	☺
Xu (2020)	☹	☹	☺	☺		☺	☺	☺
Yongchen (2020)	?	☹	☺	☺		☺	☺	☺
Zeng (2020)	☹	☹	☺	☺		☹	☺	☺
Zhang (2020)	☹	☺	☺	☺		☺	☺	☺
Zhao (2020)	☹	☹	☺	☺		☺	☺	☺
Zhong (2020)	?	☺	☺	☺		?	☺	☺

☺ = low risk; ☹ = high risk; ? = unclear risk

Table S4. Summary of all 55 included studies across 39 publications.

First author (year)	Country	Study design/ type	No. of samples (total/ COVID-19/ healthy/ other pathogen samples)	Type of immunoassay	Commercial name	Type of antibody tests (antigen)	Rapid test
Bryan (2020)[29]	United States	Case control/ retrospective	1709/689/1020/0	CLIA	Abbott Architect SARS-CoV-2 IgG Assay	IgG (N)	No
Cai (2020)[30]	China	Case control/ retrospective	643/276/200/167	CLIA	NR	IgM (N)/ IgG (N)	No
Cassaniti (2020)[31]	Italy	Cohort/ prospective	50/38/12/0	LFIA	VivaDiag COVID-19 IgM/IgG	IgM (N, S)/ IgG (N, S)	Yes
Cassaniti (2020)[31]	Italy	Case control/ retrospective	60/30/20/10	LFIA	VivaDiag COVID-19 IgM/IgG	IgM (N, S)/ IgG (N, S)	Yes
Chen (2020)[32]	China	Case control/ retrospective	19/7/12/0	LFIA	NR	IgG (N)	No
Demey (2020)[33]	France	Case control/ retrospective	26/22/0/4	GICA	Biotime Biotechnology Co	IgM (NR)/ IgG (NR)	No
Demey (2020)[33]	France	Case control/ retrospective	33/22/0/11	LFIA	Autobio Diagnostics Co	IgM (NR)/ IgG (NR)	No
Demey (2020)[33]	France	Case control/ retrospective	29/22/0/7	ICA	ISIA BioTechnology Co	IgM (NR)/ IgG (NR)	No
Demey (2020)[33]	France	Case control/ retrospective	26/22/0/4	ICA	Biolidics	IgM (NR)/ IgG (NR)	No
Dohla (2020)[34]	Germany	Cohort/ prospective	49/22/27/0	NR	NR	IgM (NR)/ IgG (NR)	Yes
Du (2020)[35]	China	Case series/ retrospective	60/60/0/0	NR	NR	IgM (N, S)/ IgG (N, S)	No

Fu (2020)[36]	China	Case series/ retrospective	5/5/0/0	NR	NR	IgM (NR)/ IgG (NR)	No
Gao (2020)[37]	China	Case series/ retrospective	37/37/0/0	CLIA	NR	IgM (N, S)/ IgG (N, S)	No
Gao (2020)[37]	China	Case series/ retrospective	37/37/0/0	GICA	NR	IgM (N, S)/ IgG (N, S)	No
Gao (2020)[37]	China	Case series/ retrospective	37/37/0/0	ELISA	NR	IgM (N, S)/ IgG (N, S)	No
Guo (2020)[38]	China	Case control/ retrospective	493/108/100/285	ELISA	NR	IgM (N)	No
Guo (2020)[38]	China	Cohort/ prospective	6/2/4/0	ELISA	NR	IgM (N)	No
Hoffman (2020)[39]	Sweden	Case control/ retrospective	153/29/124/0	ICA	COVID-19 IgG/IgM Rapid Test Cassette (whole blood/serum/plasma), Product/Model: GCCOV-402a, Lot: 2003242, Zhejiang Orient Gene Biotech Co Ltd, Huzhou, Zhejiang, China)	IgM (NR)/ IgG (NR)	No
Hou (2020)[40]	China	Case series/ retrospective	338/338/0/0	CLIA	YHLO-CLIA-IgG, YHLO-CLIA-IgM by YHLO Biotech Co Ltd Shen Zhen, China	IgM (N, S)/ IgG (N, S)	No
Imai (2020)[41]	Japan	Case control/ retrospective	187/139/48/0	NR	One Step Novel Coronavirus (COVID-19) IgM/IgG Antibody Test	IgM (NR)/ IgG (NR)	No
Infantino (2020)[42]	Italy	Case control/ retrospective	128/64/64/0	CLIA	iFlash1800 CLIA analyzer, Shenzhen YHLO Biotech Co Ltd	IgM (N, S)/ IgG (N, S)	No

Jin (2020)[43]	China	Case control/ retrospective	76/43/33/0	CLIA	Shenzhen YHLO Biotech Co Ltd	IgM (N, S)/IgG (N, S)	No
Lee (2020)[44]	Taiwan	Case control/ retrospective	61/33/25/3	LFIA	ALLTEST 2019-nCoV IgG/IgM Rapid Test Cassette	IgM (N)/IgG (N)	Yes
Li (2020)[45]	China	Case control/ retrospective	525/397/128/0	LFIA	NR	IgM (S)/ IgG (S)	Yes
Liu (2020)[46]	China	Case control/ retrospective	314/214/100/0	ELISA	rN protein-based ELISA kit (Lizhu, Zhuhai, China)	IgM (N, S)/ IgG (N, S)	No
Liu (2020)[46]	China	Case control/ retrospective	314/214/100/0	ELISA	rS-based ELISA kit (Hotgen, Beijing, China)	IgM (N, S)/ IgG (N, S)	No
Long (2020)[47]	China	Case series/ prospective	415/363/52/0	CLIA	Magnetic chemiluminescence enzyme immunoassay (MCLIA)	IgM (N, S)/ IgG (N, S)	No
Long (2020)[47]	China	Cohort/prospective	164/16/148/0	CLIA	Magnetic chemiluminescence enzyme immunoassay (MCLIA)	IgM (N, S)/ IgG (N, S)	No
Padoan (2020a)[48]	Italy	Case series/ retrospective	151/151/0/0	CLIA	MAGLUMI 2000 Plus	IgM (N, S)	No
Padoan (2020a)[48]	Italy	Case series/ retrospective	67/67/0/0	ELISA	Euroimmun Medizinische Laboradiagnostika, Luebeck, German	IgA (N, S)	No
Padoan (2020b)[49]	Italy	Case series/ retrospective	87/87/0/0	CLIA	MAGLUMI 2019-nCov IgM and 2019- nCov IgG (CLIA) systems	IgM (N, S)/ IgG (N, S)	No
Pan (2020)[50]	China	Case series/ retrospective	125/86/39/0	GICA	Colloidal gold-based immunochromatographic (ICG) strip	IgM (N, S)/ IgG (N, S)	Yes
Perera (2020)[51]	Hong Kong	Case control/ retrospective	258/51/200/7	ELISA	NR	IgM (S)/ IgG (S)	No

Qu (2020)[52]	China	Case control/ retrospective	385/347/28/10	CLIA	iFlash-SARS- CoV-2 IgG/IgM chemiluminescent immunoassay kit (C86095G/C86095M, YHLO BIOTECH, Shenzhen)	IgM (N, S)/ IgG (N, S)	No
Shen (2020)[53]	China	Cohort/ retrospective	150/97/53/10	GICA	Colloidal gold immunochromatography antibody detection kit (Shanghai Outdo Biotech Co. Ltd, China, LOT: 20200101)	IgM (M, N, S)/ IgG (M, N, S)	Yes
Shi (2020)[54]	China	Case series/ retrospective	114/114/0/0	Dry fluorescence	Dry fluorescence immunoassay (Lansionbio, China)	IgM (NR)	No
Solodky (2020)[55]	France	Cohort/ retrospective	85/10/75/0	LFIA	Toda Coronadiag® (TODA Pharma, Strasbourg France). Coronadiag®	IgM (NR)/ IgG (NR)	Yes
Solodky (2020)[55]	France	Cohort/ retrospective	244/14/230/0	LFIA	Toda Coronadiag® (TODA Pharma, Strasbourg France). Coronadiag®	IgM (NR)/ IgG (NR)	Yes
Spicuzza (2020)[56]	Italy	Case control/ prospective	37/23/14/0	ICA	2019-nCoV IgG/IgM Antibody Rapid Test Kit (Beijing Diagreat Biotechnologies Co., Ltd)	IgM (NR)/ IgG (NR)	Yes
To (2020)[57]	Hong Kong	Case series/ prospective	108/108/0/0	ELISA	NR	IgM (N, S)/ IgG (N, S)	No
To (2020)[57]	Hong Kong	Case series/ prospective	108/108/0/0	ELISA	NR	IgM (N, S)/ IgG (N, S)	No
Wang (2020)[58]	China	Case series/ prospective	72/72/0/0	NR	"New Coronavirus (2019-nCoV) Antibody Detection Kit" (INNOVITA, China)	IgM (NR)/ IgG (NR)	Yes
Wu (2020)[10]	China	Cohort/ prospective	381/1/380/0	NR	(Beijing Innovita Biological Technology Co., Ltd., 2019-nCoV Ab Test (Colloidal Gold))	IgM (NR)/ IgG (NR)	Yes

Wu (2020)[10]	China	Cohort/ prospective	1021/0/1021/0	NR	(Beijing Innovita Biological Technology Co., Ltd., 2019-nCoV Ab Test (Colloidal Gold))	IgM (NR)/ IgG (NR)	Yes
Xiang (2020)[59]	China	Case control/ prospective	276/216/60/0	ELISA	ELISA kits, Livzon Inc, Zhuhai, P.R.China, lot number of IgM: 20200308, IgG: 20200308	IgM (N)/ IgG (N)	No
Xie (2020)[60]	China	Cohort/ retrospective	56/16/40/0	CLIA	YHLO Biological Technology Co., Ltd., Shenzhen, China	IgM (E, N)/ IgG (E, N)	No
Xu (2020)[61]	China	Cohort/ prospective	6/2/4/0	GICA	SARS-CoV-2-specific immunoglobin M (IgM) screening testing by gold immunochromatography assay (Hotgen Biotech Co., Ltd., Beijing, China)	IgM (NR)/ IgG (NR)	No
Xu (2020)[61]	China	Cohort/ prospective	6/2/4/0	ELISA	ELISA, developed by Institute of Pathogen Biology, Chinese Academy of Medical Sciences & Peking Union Medical College	IgM (NR)/ IgG (NR)	No
Yongchen (2020)[62]	China	Case series/ retrospective	91/91/0/0	GICA	gold immunochromatography assay supplied by Innovita Co., LTd, China	IgM (N, S)/ IgG (N, S)	Yes
Zeng (2020)[63]	China	Case series/ retrospective	6/6/0/0	NR	NR	IgM (N, S)/ IgG (N, S)	No
Zhang (2020)[64]	China	Case series/ retrospective	112/112/0/0	ELISA	IgM and IgG antibody detection kit (Yahuilong Biotechnology, Shenzhen, China)	IgM (E, N)/ IgG (E, N)	No
Zhao (2020)[65]	China	Case series/ prospective	535/535/0/0	ELISA	(ELISA) kits supplied by Beijing Wantai Biological Pharmacy Enterprise Co.,Ltd,	IgM (S)/ IgG (N)	No

Zhong (2020)[66]	China	Case control/ prospective	347/47/300/0	ELISA	NR	IgM (N)/ IgG (N)	No
Zhong (2020)[66]	China	Case control/ prospective	347/47/300/0	ELISA	NR	IgM (N)/ IgG (N)	No
Zhong (2020)[66]	China	Case control/ prospective	347/47/300/0	CLIA	NR	IgM (N)/ IgG (N)	No

CLIA, Chemiluminescent immunoassay. ELISA, Enzyme-linked immunosorbent assay. GICA, Gold immunochromatographic assay.
ICA, Immunochromatographic assay. LFIA, Lateral flow immunochromatographic assay. E, Envelope protein. M, Membrane protein. N, Nucleocapsid protein. S, Spike protein. NR, Not reported.

Table S5. Summary of diagnostic performance for all included studies using IgM testing.

Time of IgM test and First author (year)	TP	FN	FP	TN	Prevalence (95% CI)	Positive Predictive Value (95% CI)	Negative Predictive Value (95% CI)	F1 score
By day 7								
Demey (2020)[33]	9	13	0	4	0.846 (0.651 – 0.956)	1	0.235 (0.179 – 0.303)	0.581
Demey (2020)[33]	10	12	0	11	0.667 (0.482 – 0.820)	1	0.478 (0.385 – 0.573)	0.625
Demey (2020)[33]	5	17	0	7	0.759 (0.565 – 0.897)	1	0.292 (0.247 – 0.341)	0.370
Demey (2020)[33]	9	13	0	4	0.846 (0.651 – 0.956)	1	0.235 (0.179 – 0.303)	0.581
Imai (2020)[41]	25	65	1	47	0.652 (0.567 – 0.731)	0.962 (0.778 – 0.994)	0.420 (0.387 – 0.453)	0.431
Liu (2020)[46]	20	18	0	100	0.275 (0.203 – 0.358)	1	0.847 (0.799 – 0.886)	0.690
Liu (2020)[46]	19	19	0	100	0.275 (0.203 – 0.358)	1	0.840 (0.793 – 0.879)	0.667
Long (2020)[47]	18	27	3	49	0.464 (0.362 – 0.568)	0.857 (0.654 – 0.950)	0.645 (0.586 – 0.699)	0.545
Pan (2020)[50]	3	24	9	30	0.409 (0.290 – 0.537)	0.250 (0.090 – 0.528)	0.556 (0.501 – 0.608)	0.154
Perera (2020)[51]	3	3	3	204	0.028 (0.010 – 0.060)	0.500 (0.201 – 0.799)	0.986 (0.968 – 0.993)	0.500
Xiang (2020)[59]	4	5	0	60	0.130 (0.061 – 0.233)	1	0.923 (0.870 – 0.976)	0.615
By day 14								
Demey (2020)[33]	21	1	0	4	0.846 (0.651 – 0.956)	1	0.800 (0.371 – 0.965)	0.977
Demey (2020)[33]	19	3	0	11	0.667 (0.482 – 0.820)	1	0.786 (0.562 – 0.913)	0.927
Demey (2020)[33]	17	5	0	7	0.759 (0.565 – 0.897)	1	0.583 (0.393 – 0.752)	0.872
Demey (2020)[33]	22	0	0	4	0.846 (0.651 – 0.956)	1	1	1
Hoffman (2020)[39]	7	3	0	124	0.075 (0.036 – 0.133)	1	0.976 (0.941 – 0.991)	0.824
Imai (2020)[41]	12	13	1	47	0.342 (0.235 – 0.463)	0.923 (0.623 – 0.989)	0.783 (0.712 – 0.841)	0.632
Infantino (2020)[42]	44	20	5	59	0.500 (0.410 – 0.590)	0.898 (0.789 – 0.954)	0.747 (0.671 – 0.810)	0.779
Jin (2020)[43]	13	14	0	33	0.450 (0.321 – 0.584)	1	0.702 (0.621 – 0.772)	0.650
Liu (2020)[46]	39	15	0	100	0.351 (0.276 – 0.432)	1	0.870 (0.813 – 0.911)	0.839
Liu (2020)[46]	45	9	0	100	0.351 (0.276 – 0.432)	1	0.917 (0.860 – 0.953)	0.909
Long (2020)[47]	55	15	3	49	0.574 (0.481 – 0.663)	0.948 (0.859 – 0.982)	0.766 (0.675 – 0.837)	0.859
Pan (2020)[50]	22	6	9	30	0.418 (0.299 – 0.545)	0.710 (0.572 – 0.817)	0.833 (0.707 – 0.912)	0.746
Perera (2020)[51]	13	1	3	204	0.063 (0.035 – 0.104)	0.813 (0.583 – 0.931)	0.995 (0.969 – 0.999)	0.867
Xiang (2020)[59]	20	4	0	60	0.286 (0.192 – 0.395)	1	0.938 (0.860 – 0.974)	0.909
By day 21								
Demey (2020)[33]	22	0	0	4	0.846 (0.651 – 0.956)	1	1	1
Demey (2020)[33]	19	3	0	11	0.667 (0.482 – 0.820)	1	0.786 (0.562 – 0.913)	0.927
Demey (2020)[33]	19	3	0	7	0.759 (0.565 – 0.897)	1	0.700 (0.449 – 0.870)	0.927
Demey (2020)[33]	22	0	0	4	0.846 (0.651 – 0.956)	1	1	1
Hoffman (2020)[39]	13	6	0	124	0.133 (0.082 – 0.200)	1	0.954 (0.914 – 0.976)	0.813
Imai (2020)[41]	23	1	1	47	0.333 (0.227 – 0.454)	0.958 (0.768 – 0.994)	0.979 (0.873 – 0.997)	0.958

Liu (2020)[46]	45	10	0	100	0.355 (0.280 – 0.436)	1	0.909 (0.851 – 0.946)	0.900
Liu (2020)[46]	53	2	0	100	0.355 (0.280 – 0.436)	1	0.980 (0.928 – 0.995)	0.981
Long (2020)[47]	16	1	3	49	0.246 (0.151 – 0.365)	0.842 (0.639 – 0.942)	0.980 (0.880 – 0.997)	0.889
Xiang (2020)[59]	14	7	0	60	0.259 (0.168 – 0.369)	1	0.896 (0.824 – 0.940)	0.800
By day 28								
Liu (2020)[46]	26	6	0	100	0.242 (0.172 – 0.325)	1	0.943 (0.890 – 0.972)	0.897
Liu (2020)[46]	28	4	0	100	0.242 (0.172 – 0.325)	1	0.962 (0.909 – 0.984)	0.933
Perera (2020)[51]	9	2	3	204	0.050 (0.026 – 0.089)	0.750 (0.485 – 0.905)	0.990 (0.967 – 0.997)	0.783
Xiang (2020)[59]	4	3	0	60	0.104 (0.043 – 0.204)	1	0.952 (0.895 – 0.979)	0.727
After day 28								
Liu (2020)[46]	9	4	0	100	0.115 (0.063 – 0.189)	1	0.962 (0.917 – 0.983)	0.818
Liu (2020)[46]	12	1	0	100	0.115 (0.063 – 0.189)	1	0.990 (0.938 – 0.999)	0.960
Perera (2020)[51]	12	0	3	204	0.055 (0.029 – 0.094)	0.800 (0.565 – 0.925)	1	0.889
Xiang (2020)[59]	12	2	0	60	0.189 (0.108 – 0.297)	1	0.968 (0.893 – 0.991)	0.923

Table S6. Summary of diagnostic performance for all included studies using IgG testing.

Time of IgG test and First author (year)	TP	FN	FP	TN	Prevalence (95% CI)	Positive Predictive Value (95% CI)	Negative Predictive Value (95% CI)	F1 score
By day 7								
Demey (2020)[33]	9	13	0	4	0.846 (0.651 – 0.956)	1	0.235 (0.179 – 0.303)	0.581
Demey (2020)[33]	10	12	1	10	0.667 (0.482 – 0.820)	0.909 (0.594 – 0.986)	0.455 (0.353 – 0.560)	0.606
Demey (2020)[33]	5	17	0	7	0.759 (0.565 – 0.897)	1	0.292 (0.247 – 0.341)	0.370
Demey (2020)[33]	9	13	0	4	0.846 (0.651 – 0.956)	1	0.235 (0.179 – 0.303)	0.581
Imai (2020)[41]	3	87	0	48	0.652 (0.567 – 0.731)	1	0.356 (0.347 – 0.364)	0.065
Liu (2020)[46]	15	23	0	100	0.275 (0.203 – 0.358)	1	0.813 (0.771 – 0.849)	0.566
Liu (2020)[46]	19	19	0	100	0.275 (0.203 – 0.358)	1	0.840 (0.793 – 0.879)	0.667
Long (2020)[47]	18	27	3	49	0.464 (0.362 – 0.568)	0.857 (0.654 – 0.950)	0.645 (0.586 – 0.699)	0.545
Pan (2020)[50]	1	26	15	24	0.409 (0.290 – 0.537)	0.063 (0.009 – 0.322)	0.480 (0.416 – 0.545)	0.047
Perera (2020)[51]	3	3	0	207	0.028 (0.010 – 0.063)	1	0.986 (0.969 – 0.994)	0.667
Xiang (2020)[59]	4	5	3	57	0.130 (0.061 – 0.233)	0.571 (0.262 – 0.834)	0.919 (0.864 – 0.954)	0.500
By day 14								
Demey (2020)[33]	21	1	0	4	0.846 (0.651 – 0.956)	1	0.800 (0.371 – 0.965)	0.977
Demey (2020)[33]	22	0	1	10	0.667 (0.482 – 0.820)	0.957 (0.773 – 0.993)	1	0.978
Demey (2020)[33]	20	2	0	7	0.759 (0.565 – 0.897)	1	0.778 (0.483 – 0.929)	0.952
Demey (2020)[33]	22	0	0	4	0.846 (0.651 – 0.956)	1	1	1
Hoffman (2020)[39]	9	1	1	123	0.075 (0.036 – 0.133)	0.900 (0.558 – 0.985)	0.992 (0.950 – 0.999)	0.900
Imai (2020)[41]	2	23	0	48	0.342 (0.235 – 0.463)	1	0.676 (0.650 – 0.701)	0.148
Infantino (2020)[42]	46	18	0	64	0.500 (0.410 – 0.590)	1	0.780 (0.706 – 0.840)	0.836
Jin (2020)[43]	24	3	3	30	0.450 (0.321 – 0.584)	0.889 (0.730 – 0.960)	0.909 (0.774 – 0.967)	0.889
Liu (2020)[46]	39	15	0	100	0.351 (0.276 – 0.432)	1	0.870 (0.813 – 0.911)	0.839
Liu (2020)[46]	41	13	0	100	0.351 (0.276 – 0.432)	1	0.885 (0.827 – 0.925)	0.863
Long (2020)[47]	63	7	3	49	0.574 (0.481 – 0.663)	0.955 (0.875 – 0.984)	0.875 (0.776 – 0.934)	0.926
Pan (2020)[50]	16	12	15	24	0.418 (0.299 – 0.545)	0.516 (0.390 – 0.640)	0.667 (0.550 – 0.766)	0.542
Perera (2020)[51]	10	4	0	207	0.063 (0.035 – 0.104)	1	0.981 (0.958 – 0.992)	0.833
Xiang (2020)[59]	20	4	3	57	0.286 (0.192 – 0.395)	0.870 (0.686 – 0.953)	0.934 (0.853 – 0.966)	0.851
By day 21								
Demey (2020)[33]	22	0	0	4	0.846 (0.651 – 0.956)	1	1	1
Demey (2020)[33]	22	0	1	10	0.667 (0.482 – 0.820)	0.957 (0.773 – 0.993)	1	0.978
Demey (2020)[33]	22	0	0	7	0.759 (0.565 – 0.897)	1	1	1
Demey (2020)[33]	22	0	0	4	0.846 (0.651 – 0.956)	1	1	1
Hoffman (2020)[39]	18	1	1	123	0.133 (0.082 – 0.200)	0.947 (0.718 – 0.992)	0.992 (0.948 – 0.999)	0.947
Imai (2020)[41]	15	9	0	48	0.333 (0.227 – 0.454)	1	0.842 (0.761 – 0.899)	0.769

Liu (2020)[46]	48	7	0	100	0.355 (0.280 – 0.436)	1	0.935 (0.877 – 0.966)	0.932
Liu (2020)[46]	51	4	0	100	0.355 (0.280 – 0.436)	1	0.962 (0.907 – 0.985)	0.962
Long (2020)[47]	17	0	3	49	0.246 (0.151 – 0.365)	0.850 (0.654 – 0.944)	1	0.919
Xiang (2020)[59]	17	4	3	57	0.259 (0.168 – 0.369)	0.850 (0.649 – 0.946)	0.934 (0.855 – 0.972)	0.829
By day 28								
Liu (2020)[46]	28	4	0	100	0.242 (0.172 – 0.325)	1	0.962 (0.909 – 0.984)	0.933
Liu (2020)[46]	27	5	0	100	0.242 (0.172 – 0.325)	1	0.952 (0.899 – 0.978)	0.915
Perera (2020)[51]	9	2	0	207	0.050 (0.026 – 0.089)	1	0.990 (0.967 – 0.997)	0.900
Xiang (2020)[59]	4	3	3	57	0.104 (0.043 – 0.204)	0.571 (0.271 – 0.827)	0.950 (0.890 – 0.978)	0.571
After day 28								
Liu (2020)[46]	13	0	0	100	0.115 (0.063 – 0.189)	1	1	1
Liu (2020)[46]	12	1	0	100	0.115 (0.062 – 0.189)	1	0.990 (0.938 – 0.999)	0.960
Perera (2020)[51]	12	0	0	207	0.055 (0.029 – 0.094)	1	1	1
Xiang (2020)[59]	14	0	3	57	0.189 (0.108 – 0.297)	0.824 (0.608 – 0.934)	1	0.903

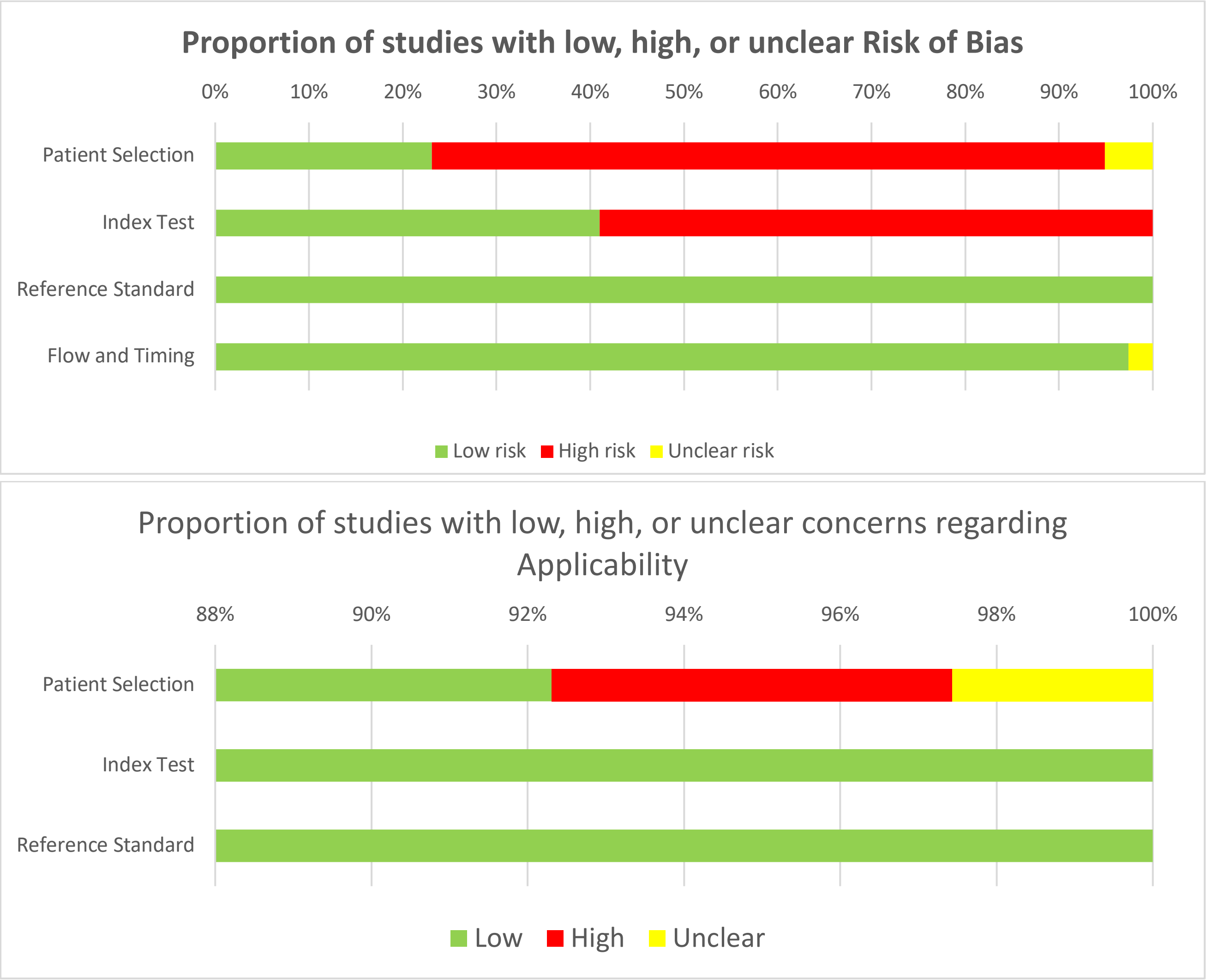

Figure S1. Bar charts demonstrating quality of included studies assessed using QUADAS-2.

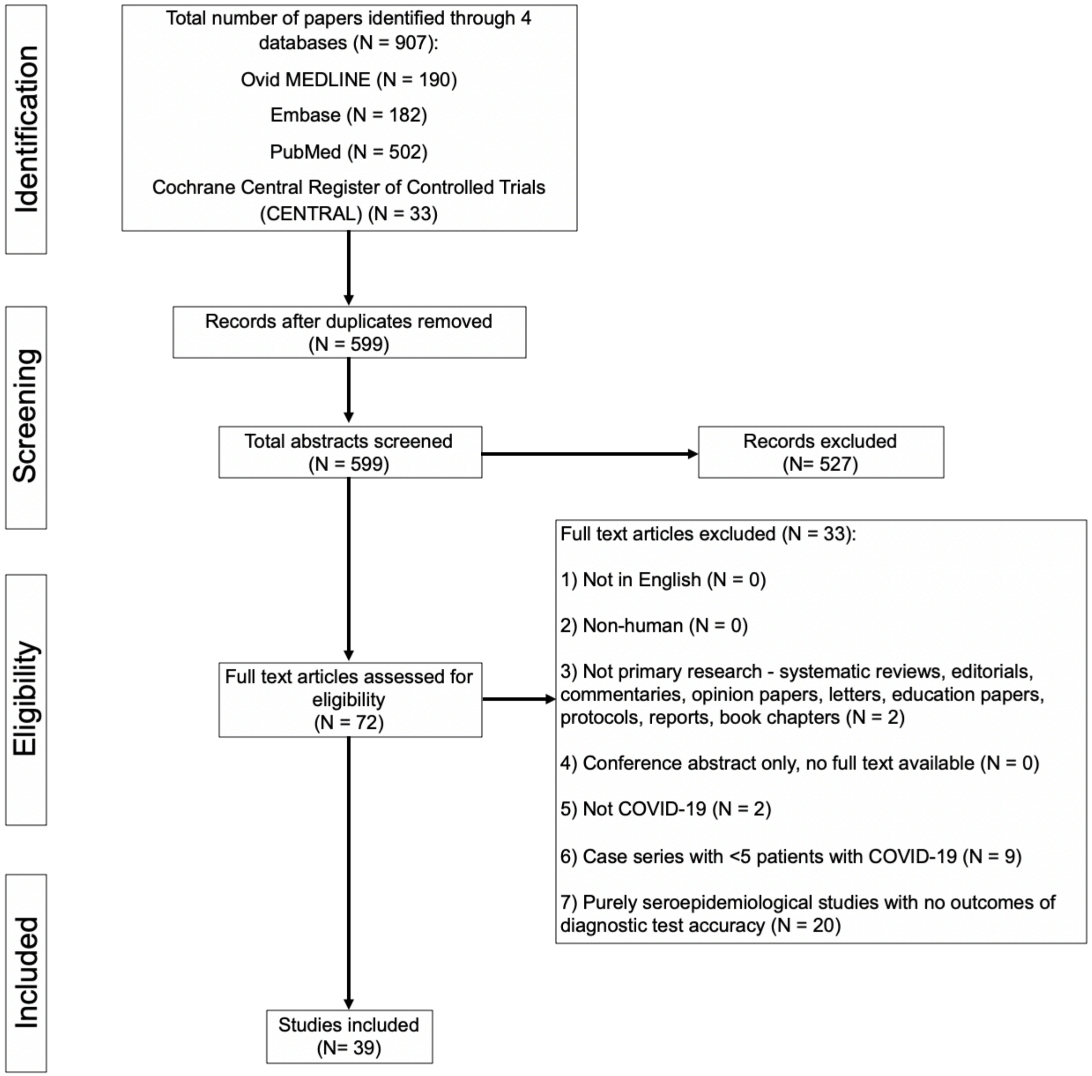

Figure S2. PRISMA flow diagram showing study selection and inclusion.

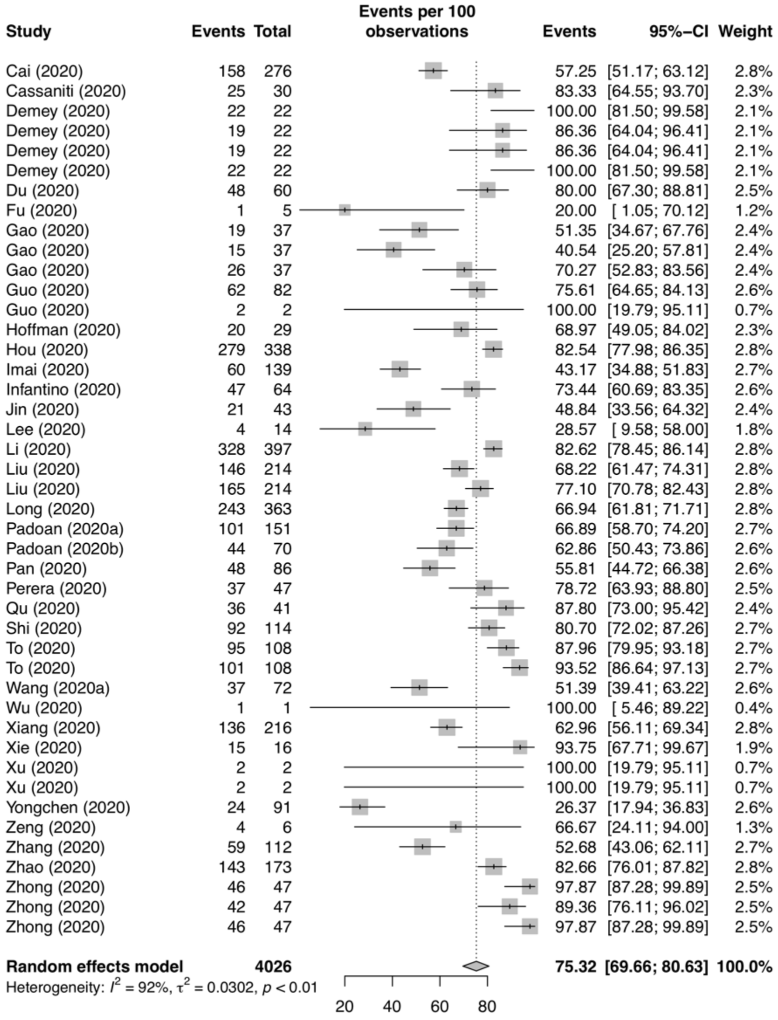

Figure S3. Pooled percentage of IgM seroconversion across all included studies.

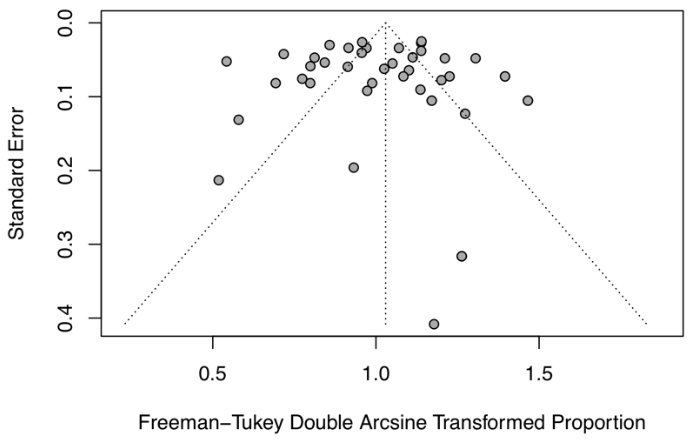

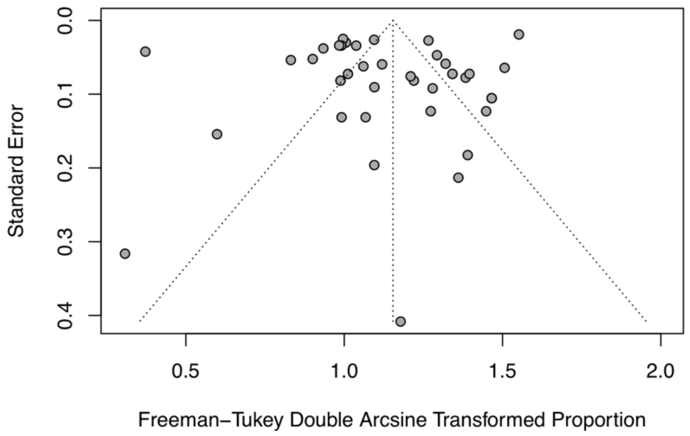

Figure S4. Funnel plots studies reporting IgM seroconversion (top) and IgG seroconversion (bottom).

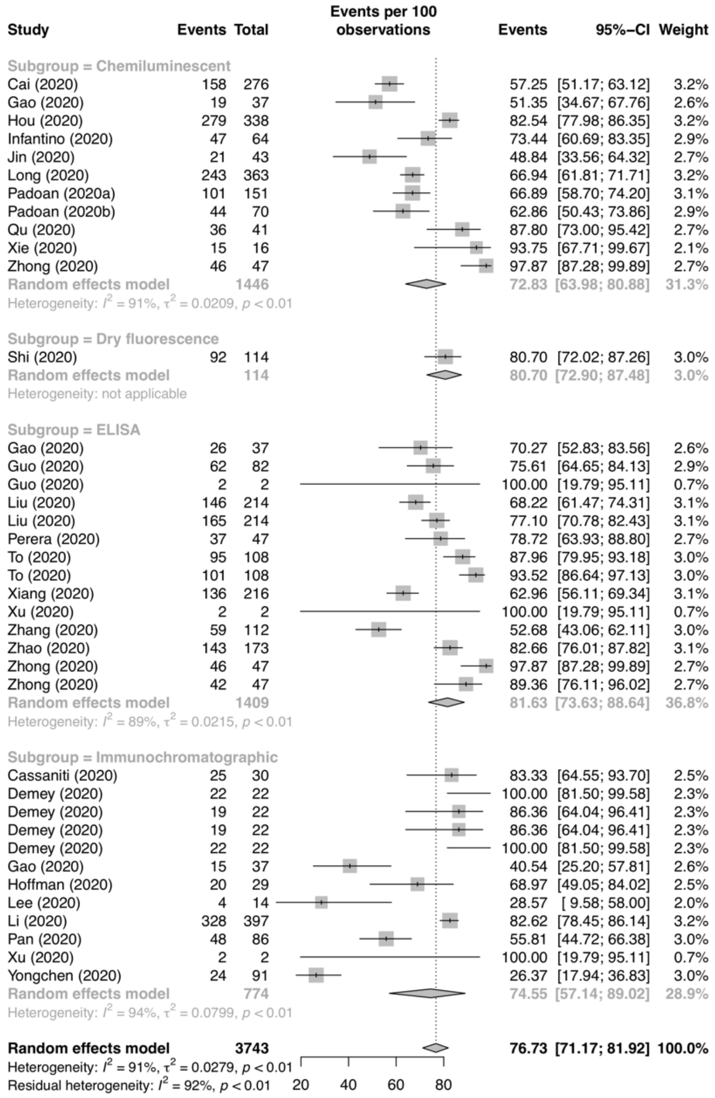

Figure S5. Subgroup analysis of IgM seroconversion percentages across studies stratified by type of immunoassay.

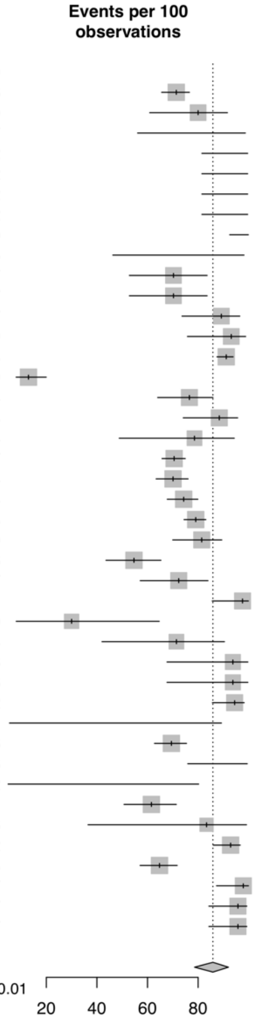

Study	Events	Total	Events	95%–CI	Weight
Bryan (2020)	689	689	100.00	[99.31; 99.99]	2.6%
Cai (2020)	197	276	71.38	[65.59; 76.56]	2.6%
Cassaniti (2020)	24	30	80.00	[60.87; 91.60]	2.4%
Chen (2020)	7	7	100.00	[56.09; 98.68]	1.8%
Demey (2020)	22	22	100.00	[81.50; 99.58]	2.3%
Demey (2020)	22	22	100.00	[81.50; 99.58]	2.3%
Demey (2020)	22	22	100.00	[81.50; 99.58]	2.3%
Demey (2020)	22	22	100.00	[81.50; 99.58]	2.3%
Du (2020)	60	60	100.00	[92.50; 99.85]	2.5%
Fu (2020)	5	5	100.00	[46.29; 98.13]	1.6%
Gao (2020)	26	37	70.27	[52.83; 83.56]	2.4%
Gao (2020)	26	37	70.27	[52.83; 83.56]	2.4%
Gao (2020)	33	37	89.19	[73.64; 96.48]	2.4%
Hoffman (2020)	27	29	93.10	[75.79; 98.80]	2.4%
Hou (2020)	308	338	91.12	[87.44; 93.84]	2.6%
Imai (2020)	18	139	12.95	[8.06; 19.95]	2.6%
Infantino (2020)	49	64	76.56	[64.02; 85.88]	2.5%
Jin (2020)	38	43	88.37	[74.12; 95.64]	2.5%
Lee (2020)	11	14	78.57	[48.82; 94.29]	2.1%
Li (2020)	280	397	70.53	[65.73; 74.92]	2.6%
Liu (2020)	150	214	70.09	[63.41; 76.05]	2.6%
Liu (2020)	159	214	74.30	[67.81; 79.90]	2.6%
Long (2020)	287	363	79.06	[74.44; 83.06]	2.6%
Padoan (2020b)	57	70	81.43	[69.98; 89.36]	2.5%
Pan (2020)	47	86	54.65	[43.59; 65.29]	2.6%
Perera (2020)	34	47	72.34	[57.13; 83.91]	2.5%
Qu (2020)	40	41	97.56	[85.59; 99.87]	2.5%
Solodky (2020)	3	10	30.00	[8.09; 64.63]	2.0%
Solodky (2020)	10	14	71.43	[42.00; 90.42]	2.1%
To (2020)	15	16	93.75	[67.71; 99.67]	2.2%
To (2020)	15	16	93.75	[67.71; 99.67]	2.2%
Wang (2020a)	68	72	94.44	[85.65; 98.21]	2.5%
Wu (2020)	1	1	100.00	[5.46; 89.22]	0.8%
Xiang (2020)	150	216	69.44	[62.77; 75.42]	2.6%
Xie (2020)	16	16	100.00	[75.93; 99.43]	2.2%
Xu (2020)	0	2	0.00	[4.89; 80.21]	1.1%
Yongchen (2020)	56	91	61.54	[50.72; 71.37]	2.6%
Zeng (2020)	5	6	83.33	[36.48; 99.12]	1.7%
Zhang (2020)	104	112	92.86	[85.98; 96.64]	2.6%
Zhao (2020)	112	173	64.74	[57.08; 71.74]	2.6%
Zhong (2020)	46	47	97.87	[87.28; 99.89]	2.5%
Zhong (2020)	45	47	95.74	[84.27; 99.26]	2.5%
Zhong (2020)	45	47	95.74	[84.27; 99.26]	2.5%
**Random effects model**		**4211**	**85.85**	**[78.61; 91.99]**	**100.0%**

Heterogeneity: $I^2 = 96\%$, $\tau^2 = 0.0722$, $p < 0.01$

Figure S6. Pooled percentage of IgG seroconversion across all included studies.

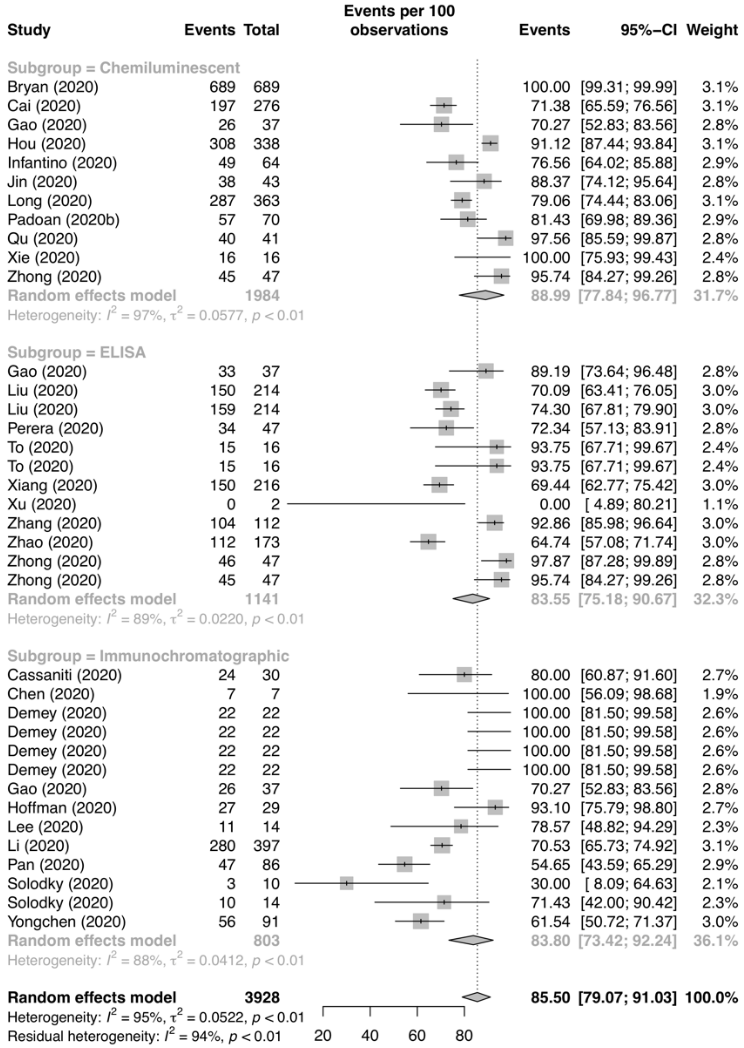

Figure S7. Subgroup analysis of IgG seroconversion percentages across studies stratified by type of immunoassay.

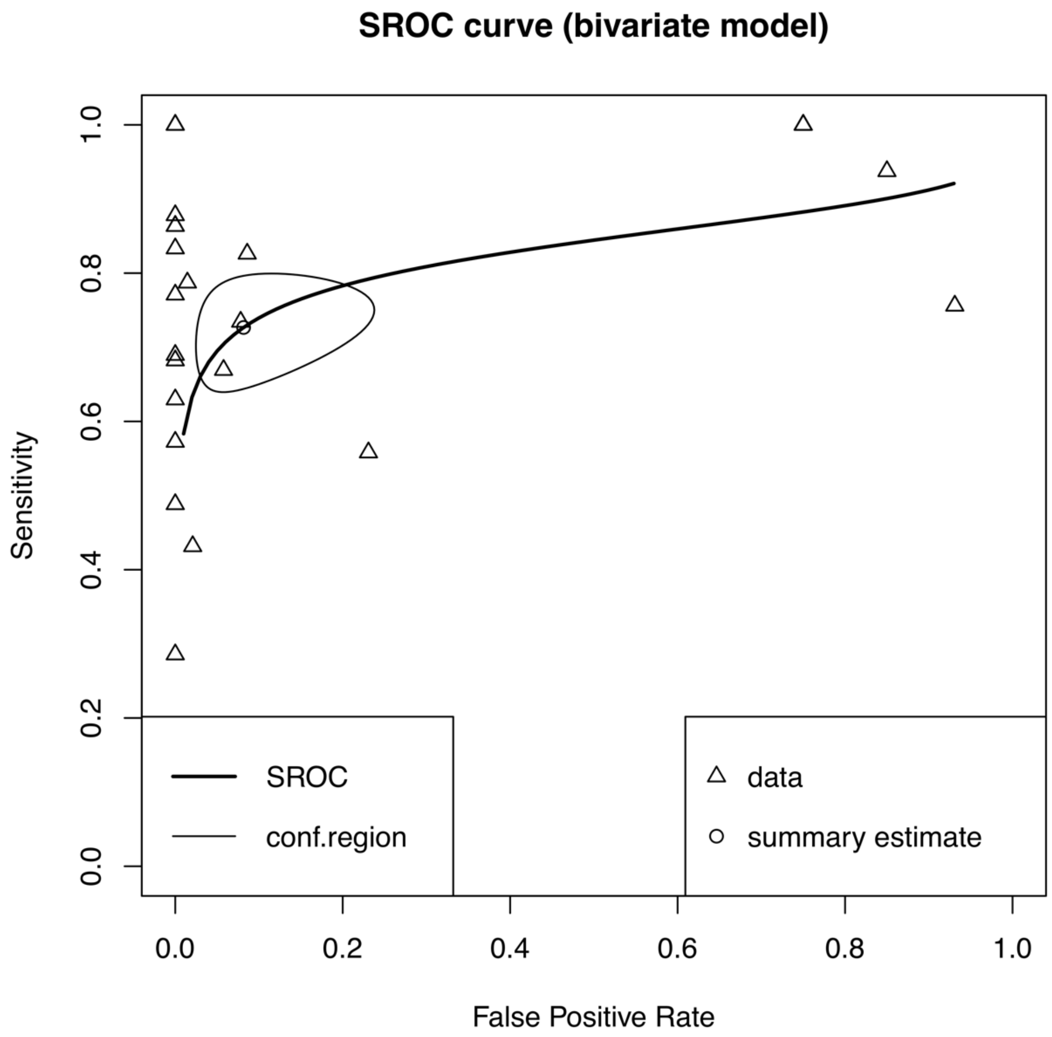

Figure S8. Bivariate summary receiver operating characteristic curve for IgM assay.

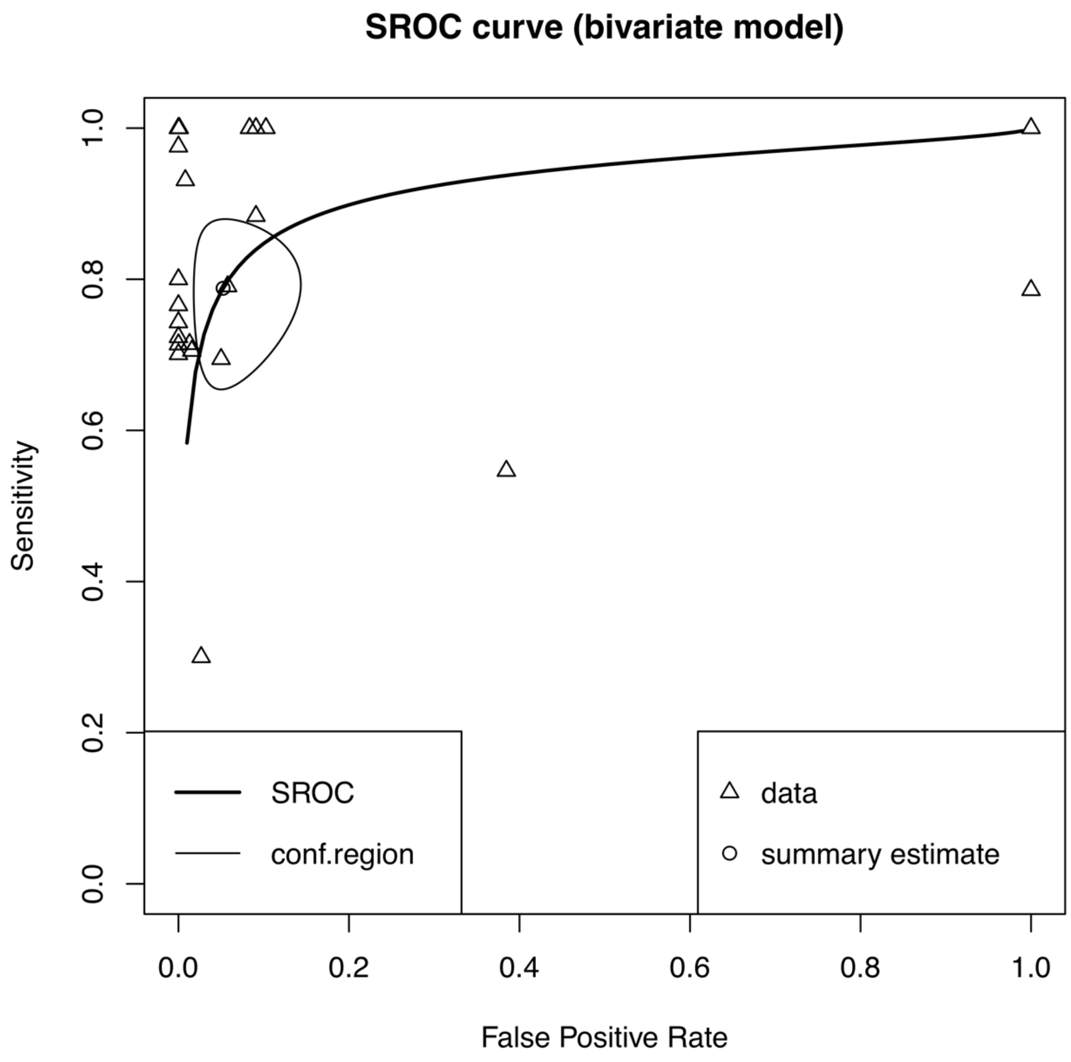

Figure S9. Bivariate summary receiver operating characteristic curve for IgG assay.

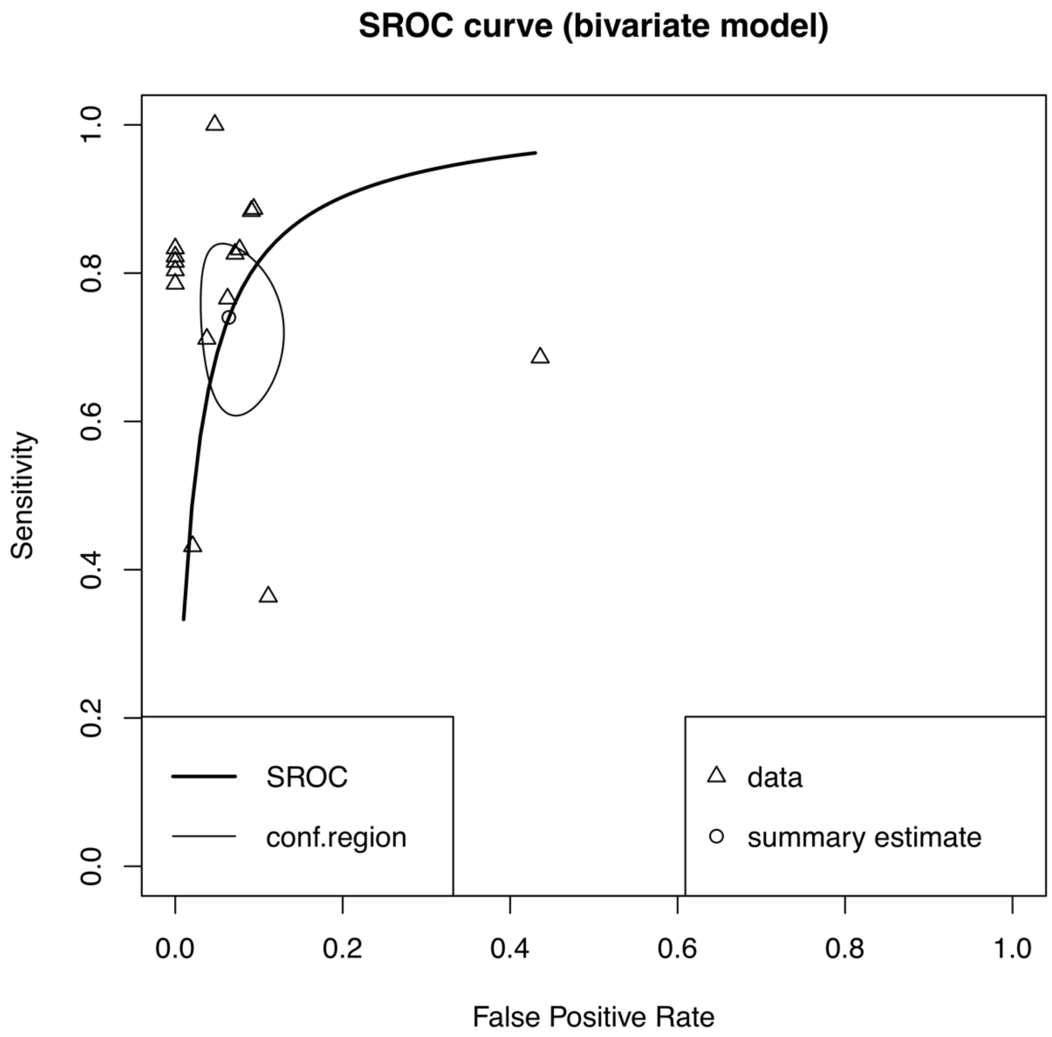

Figure S10. Bivariate summary receiver operating characteristic curve for testing using IgM or IgG.
